# Fast Bayesian Functional Principal Components Analysis

**DOI:** 10.1080/10618600.2025.2592768

**Published:** 2026-02-11

**Authors:** Joseph Sartini, Xinkai Zhou, Elizabeth Selvin, Scott Zeger, Ciprian M. Crainiceanu

**Affiliations:** a Department of Biostatistics, Johns Hopkins University, Baltimore, MD; b Department of Epidemiology, Johns Hopkins University, Baltimore, MD

**Keywords:** Bayesian methods, Functional data, Semiparametric methods, Uncertainty quantification

## Abstract

Functional Principal Components Analysis (FPCA) is a widely used analytic tool for dimension reduction of functional data. Traditional implementations of FPCA estimate the principal components from the data, then treat these estimates as fixed in subsequent analyses. To account for the uncertainty of PC estimates, we propose FAST, a fully-Bayesian FPCA with three core components: (1) projection of eigenfunctions onto an orthonormal spline basis; (2) efficient sampling of the orthonormal spline coefficient matrix using a parameter expansion scheme based on polar decomposition; and (3) ordering eigenvalues during sampling. Extensive simulation studies show that FAST is very stable and performs better compared to existing methods. FAST is motivated by and applied to a study of the variability in mealtime glucose from the Dietary Approaches to Stop Hypertension for Diabetes Continuous Glucose Monitoring (DASH4D CGM) study. All relevant STAN code and simulation routines are available as supplementary material.

## 1. Introduction

Functional principal component analysis (FPCA) is a common analytic approach for high-dimensional applications. FPCA approximates the covariance between all possible pairs of observations by identifying a small set of orthonormal principal components (FPCs). Using FPCs to provide a reduced-rank representation of functional data, rather than fixed (spline) bases (Shi et al. 1996; Rice and Wu 2001), provides a data-driven representation based on the leading modes of variability (James et al. 2000). While the means of estimating these FPCs can vary, e.g., polynomial smoothing (Yao et al. 2005), kernel smoothing (Staniswalis and Lee 1998), or sandwich covariance smoothing (Xiao et al. 2016), the key idea remains consistent. Conditioning on the FPCs, functional models become mixed effects models with a small number of random effects and inference can be conducted using existing software (Reiss and Ogden 2007; Di et al. 2009; Zhou et al. 2010; Liu and Guo 2012; Shou et al. 2015). Frequentist implementations of FPCA follow a two-step approach: (1) diagonalizing a smooth estimator of the observed functional covariance; and then (2) conducting inference conditional on the FPC estimates (Peng and Paul 2009; Crainiceanu and Goldsmith 2010; Xiao et al. 2016). This conditional approach may lead to invalid inference in smaller samples and/or when eigenfunctions explain smaller variance (Goldsmith et al. 2013). FPC forms may be falsely identified, or FPC scores with substantial uncertainty may be used in downstream regression.

There are several Bayesian FPCA implementations that model the FPCs and their scores jointly. An early fully-Bayesian FPCA was developed by Goldsmith et al. (2015), who modeled both the fixed and random effects functions using unconstrained splines and applied post-processing to obtain orthonormal FPC samples. Jauch et al. (2021) directly sampled the FPC matrix using a parameter expansion scheme based on polar decomposition. Suarez and Ghosal (2017) proposed an empirical Bayes approach based on spline expansion which models the coefficient covariance structure. A recent approach used variational inference based on message passing (Nolan et al. 2023). Boland et al. (2023) implemented Bayesian FPCA using latent factor methods, also providing useful visualization tools for posterior FPC uncertainty. In the context of sparse functional data, Ye (2024) projected the FPCs onto an orthonormal spline basis and used Gibbs sampling approach of Hoff (2009) to sample the spline weights such that FPC orthonormality was preserved. Recently Zhang et al. (2025) proposed a robust implementation based on sequential Monte Carlo designed to handle outliers and sparse observations.

In this paper, we introduce Fast Bayesian FPCA, or FAST for short, which fuses ideas introduced by Ye (2024) and Jauch et al. (2021) and expands them to create a self-contained methodology that is computationally stable, scalable, and extensible to a variety of other data structures and models. FAST leverages orthogonal spline expansion to reduce the dimension of sampled FPC parameters to the corresponding weights, which are then efficiently sampled using a parameter expansion scheme based on polar decomposition. The dimensionality reduction of the posterior space by spline expansion combined with stabilizing order constraints of FPCA ensures that the approach has excellent convergence and mixing properties while being computationally efficient. All core components of FAST are easy to implement in Bayesian software such as STAN (Carpenter et al. 2017) and can be extended to include covariates, multilevel, sparse, and multivariate data.

This research is motivated by the Dietary Approaches to Stop Hypertension for Diabetes Continuous Glucose Monitoring (DASH4D CGM) clinical trial. DASH4D CGM was a crossover feeding trial which included continuous glucose monitoring (CGM) during meals that were controlled and provided by the study. Participants visited the study site three times per week to provide blood samples and receive their assigned meals, all while wearing a CGM device. When participants consumed a meal at the study center, study staff recorded the start times. Here we focus on CGM data collected after the observed meals, with dysregulation of the corresponding glycemic response being known to be associated with adverse cardiovascular outcomes (Garber 2012; Hershon et al. 2019).

Analyses of postprandial CGM glucose with verified meal times and composition are rare, and previous studies have not considered the continuous functional measurements obtained from CGM (Röhling et al., 2019; Barua et al. 2022). This paper introduces novel statistical methods designed to quantify the five-hour CGM curves from one hour before to four hours after each meal. The main results of the trial are reported elsewhere (Fang et al. 2025; Pilla et al. 2025), so this paper focuses on the variability of the CGM trajectories by diet and not on treatment effects. Specifically, it applies FPCA to the CGM curves aggregated by participant and Multilevel FPCA (MFPCA) to the individual meal response curves. The number of participants in this study is relatively small because of participant burden and data collection cost, which may result in substantial FPCA uncertainty.

The rest of the paper is organized as follows. Section 2 presents the modeling approach for FAST. Section 3 provides the STAN code. Section 4 compares FAST with existing implementations using simulation. Section 5 demonstrates the utility of FAST in estimating FPCA for the motivating data from the DASH4D clinical trial, with appropriate uncertainty quantification. Section 6 provides a short discussion.

## 2. Methods

### 2.1. FPCA Model

The functional data structure has the form $Y_{i}(t),i=1,\ldots ,N$ for $t\in \left\{t_{1},\ldots ,t_{M}\right\}\in [0,1]$, which can be thought of as functions observed on a finite grid. Assuming that $Y_{i}(t)$ are generated by the same Gaussian Process and truncating the decomposition (Kosambi 1943; Karhunen 1947; Loève 1978) produces the standard FPCA model:

(1) $$Y_{i}(t)=\mu (t)+\sum_{k=1}^{\infty }\xi_{ik}\phi_{k}(t)\approx \mu (t)+\sum_{k=1}^{K}\xi_{ik}\phi_{k}(t)+\epsilon_{i}(t).$$

Here μ(t) is the population mean, $\phi_{k}(t)\in L^{2}([0,1])$ for k=1,…,K are orthonormal eigenfunctions of the covariance operator of $Y_{i}(t),\xi_{ik}∼N\left(0,\lambda_{k}\right),\lambda_{k}$ are the eigenvalues corresponding to $\phi_{k}(t),\epsilon_{i}(t)∼N\left(0,\sigma^{2}\right)$ is the error process, and $\epsilon_{i}(t)$ and $\xi_{ik}$ are mutually independent over i and k.

For this paper, we fix the number of FPCs, K, which controls the rank of the smooth covariance operator. Choosing K is a difficult problem discussed extensively in the literature (Yao et al. 2005; Li et al. 2013; Crainiceanu et al. 2024). For the purpose of this paper, K is chosen based on the proportion of total variability explained. This can either be estimated using frequentist FPCA or our FAST procedure using a sequence of *K*s.

Model (1) is a first line approach in the analysis of high dimensional data (Crainiceanu et al. 2024) because in many applications K≪M and model (1) provides a practical, low-dimensional approximation to the original high-dimensional data. Moreover, conditional on the FPCs, $\phi_{k}(\cdot )$, the model becomes a mixed effects model with uncorrelated random effects. In this paper we treat the eigenfunctions $\phi_{k}(t)$ as parameters, estimate them from the data, and account for their uncertainty. FAST provides a stable approach to simulating the posterior distribution of all parameters, including the eigenfunctions.

### 2.2. Orthonormal Spline Expansion

All functional components are expanded using a *Q*– dimensional orthonormal spline basis, such that Q≥K. Orthonormality is defined with respect to the scalar product on $L^{2}([0,1])$, that is, $\langle f,g\rangle =\int_{0}^{1}f(x)g(x)dx$. Denote by $B(t)=\left\{B_{1}\left(t\right),\ldots ,B_{Q}(t)\right\}$ the spline basis and by $B\in R^{M\times Q}$ the matrix representation of the splines evaluated over an *M*-dimensional grid. Each column of B corresponds to a spline basis and each row corresponds to a sampling point. We model $\mu (t)=B(t)w_{\mu }$ and $\phi_{k}(t)=B(t)\psi_{k}$, where $w_{\mu }$ and $\psi_{k}$ are *Q*-dimensional vectors of spline coefficients. In the data space, these become ${\left\{\mu \left(t_{1}\right),\ldots ,\mu \left(t_{M}\right)\right\}}^{t}=Bw_{\mu }$ and ${\left\{\phi_{k}\left(t_{1}\right),\ldots ,\phi_{k}\left(t_{M}\right)\right\}}^{t}=B\psi_{k}$.

We will show that the $\phi_{k}(t)$ functions are orthonormal if and only if $\Psi^{t}\Psi =I_{K\times K}$, where $\Psi =\left[\psi_{1}|\ldots ,|\psi_{K}\right]$ is the Q×K dimensional matrix obtained by column binding the $\psi_{k}$. Indeed, as B(t) is an orthonormal spline basis, $\left\langle B_{i}(t),B_{j}(t)\right\rangle =1$ if i=j and 0 otherwise. Therefore, $\left\langle \phi_{k}(t),\phi_{k^{\prime }}(t)\right\rangle$ is equal to

$$\int_{0}^{1}\left[B(t)\psi_{k}\right]\left[B(t)\psi_{k^{\prime }}\right]dt=\sum_{i=1}^{Q}\sum_{j=1}^{Q}\psi_{k,i}\psi_{k^{\prime },j}\;\;\left[\int_{0}^{1}B_{i}(t)B_{j}(t)dt\right]=\psi_{k}^{t}\psi_{k^{\prime }}.$$

The quantity $\psi_{k}^{t}\psi_{k^{\prime }}=1$ when $k=k^{\prime }$ and 0 otherwise if and only if Ψ is orthonormal. Therefore, the eigenfunctions $\phi_{k}(t)$ are orthonormal in $L^{2}([0,1])$ if and only if the low-dimensional spline coefficients are orthonormal in $R^{Q}$. This is a crucial point of our methodology, as the orthonormality of the eigenfunctions (in infinite or high dimensional spaces) is controlled by orthonormality of vectors in a small dimensional space. Because it is much easier to sample from a small dimensional space, this projection is one of the main reasons the FAST method is stable and computationally efficient.

In functional data analysis, orthonormality in the theoretical $L^{2}([0,1])$ functional space is often confounded with orthonormality in the $R^{M}$ observed vector space. For a fixed M and equally-spaced observations, the inner product on $R^{M}$ is the Riemann sum approximation (up to the scaling factor 1/M) of the functional inner product. However, this approximation depends on the density of the observation in the functional domain and needs to be re-adjusted when the number of sampling points is changed. FAST can be used in the vector space as well by choosing the matrix B such that $B^{t}B=I_{Q}$. The resulting FPC matrix BΨ is then a product of orthonormal matrices. To maintain robustness to different M and sampling regimes, we focus here on functional orthonormality. However, our software implementation can use vector orthormality, as well.

The basis dimension Q must be sufficiently large to capture the maximum complexity of the subject-specific functions. We follow here the standard recommendations for penalized splines and use Q∈[20,40] (Ruppert 2002), which performed well in our tests (see Supplement Section 5.2). The choice of the basis matrix B can substantially impact the computational stability and efficiency of FAST. While, in theory, any spline matrix B which is orthonormal in $L^{2}[0,1]$ could be used, in practice some approaches have better computational properties and interpretability. We choose as our basis “Splinets”, an orthonormalized B-spline basis introduced by Liu et al. (2020) to maintain temporal localization. We augment their basis with a slope and intercept, appropriately orthonormalized.

### 2.3. Priors

We add smoothing penalties on μ(t) and the FPCs $\phi_{k}(t)$ to the target posterior, which have form $\alpha \int f^{2}(t)dt+(1-\alpha )\int {\left\{f^{\prime \prime }(t)\right\}}^{2}dt$ for generic function f(⋅). This penalty, used by Goldsmith et al. (2015), contains the fixed weighting parameter α, where α=0 corresponds to the famous penalized spline prior (Craven and Wahba 1978; O’Sullivan 1986). If f(t)=B(t)θ, then the penalty is a quadratic form of θ with associated matrix $P_{\alpha }$. Supplement Section 1 covers the derivation of $P_{\alpha }$. We use α=0.1 for consistency with existing implementations, but FAST can handle any α∈[0,1]. Supplement Section 5.3 indicates robustness to reasonable choice of α. The quadratic penalties are equivalent to Normal priors when $P_{\alpha }$ is non-degenerate (Brumback et al. 1999; Ruppert et al. 2003; Wood 2006). The penalties $g_{\mu }\left(w_{\mu }\right),g_{\phi_{k}}\left(\psi_{k}\right)$ have the following forms:

$$g_{\mu }\left(w_{\mu }\right)=h_{\mu }^{\text{R}\left(P_{\alpha }\right)/2}\text{exp}\left\{-\frac{h_{\mu }}{2}w_{\mu }^{t}P_{\alpha }w_{\mu }\right\},\;g_{\phi_{k}}\left(\psi_{k}\right)=h_{k}^{\text{R}\left(P_{\alpha }\right)/2}\text{exp}\left\{-\frac{h_{k}}{2}\psi_{k}^{t}P_{\alpha }\psi_{k}\right\},$$

where $R\left(P_{\alpha }\right)$ is the rank of the penalty $P_{\alpha }$ and $h_{\mu },h_{k}>0$ are the smoothing parameters for μ(⋅) and ϕ(⋅), respectively. FAST uses separate parameters for each functional component, in-line with Ye (2024) and Goldsmith et al. (2015).

We assume that the inverse variance components, including the mean smoothing parameter, have Gamma priors with the shape and rate parameters equal to 0.001. For the FPC smoothing parameters, we choose shape parameters of 0.01 and rate parameters of equal to the trace of $P_{\alpha }/2$ plus 0.01 to ensure a proper prior (see Result 1). For discussion on the choice of Gamma priors for inverse variance components, see Crainiceanu et al. (2005); Crainiceanu and Goldsmith (2010).

Consider the joint prior $g(\Psi ,H)=g_{\psi }(\Psi |H)g_{h}(H)$ on the FPCs (Ψ) and the smoothing parameters $H=diag\left(h_{1},\ldots ,h_{K}\right)$. Here $g_{\psi }(\cdot )$ is the conditional prior of Ψ given the smoothing parameter matrix H and $g_{h}(\cdot )$ is the product of independent Gamma priors on $h_{k}$. We define the conditional prior $g_{\psi }(\Psi |H)$ as follows:

$$g_{\psi }(\text{Ψ}|H)\propto \frac{\prod_{i=1}^{K}h_{i}^{\text{R}\left(P_{\alpha }\right)/2}}{\text{Vol}\left({𝒱}_{K,Q}\right)}\times \text{exp}\left\{-\text{trace}\left(H{\text{Ψ}}^{t}P_{\alpha }\text{Ψ}/2\right)\right\},$$

where $Vol\left({𝒱}_{K,Q}\right)$ is the volume of the Stiefel manifold ${𝒱}_{K,Q}$ and “trace(*A*)” denotes the trace of the matrix *A*. The conditional density $g_{\psi }(\Psi |H)$ is a combination of a uniform and smoothing prior on individual PCs. Implementing this prior in Bayesian software is straightforward.

#### Result 1.

The prior distribution $g(\Psi ,H)=g_{\psi }(\Psi |H)g_{h}(H)$ is proper if $\beta_{\psi }>trace\left(P_{\alpha }\right)/2$, where $\beta_{\psi }$ is the rate parameter for the Gamma priors on the smoothing parameters $h_{k}$.

Result 1 provides sufficient conditions under which the joint prior g(Ψ,H) is proper; proof can be found in Supplement Section 2.

### 2.4. Polar Decomposition

The conditional posterior distributions for all parameters except Ψ have analytical forms; see Supplement Section 3. The Q×K dimensional matrix parameter Ψ is orthonormal and, hence, belongs to the Stiefel manifold (James 1976), which has a finite volume and is denoted as ${𝒱}_{K,Q}$ (Mardia and Khatri 1977; Chikuse et al. 2003). Our prior on Ψ, conditional on the smoothing parameters $\left\{h_{1},\ldots ,h_{K}\right\}$, is a prior on ${𝒱}_{K,Q}$.

#### Result 2.

The conditional posterior distribution of the eigenfunction matrix Ψ is

$$f(\text{Ψ}|\text{others})\propto \text{etr}\left[\frac{\text{Ξ}{\left(Y-1_{N}^{t}⊗Bw_{\mu }\right)}^{t}B\text{Ψ}}{\sigma^{2}}-\frac{\text{Ξ}{\text{Ξ}}^{t}{\text{Ψ}}^{t}B^{t}B\text{Ψ}}{2\sigma^{2}}-\frac{H{\text{Ψ}}^{t}P\text{Ψ}}{2}\right]\text{𝟙}\left(\text{Ψ}\in {𝒱}_{K,Q}\right),$$

where $H=diag\left(h_{1},\ldots ,h_{K}\right),\Xi$ is a N×K dimensional matrix with the (i,k) entry equal to $\xi_{ik}$, and the matrix Y is the N×M dimensional matrix with the row i equal to $Y_{i}^{t}$. The quantity $1_{N}$ is the N-dimensional column vector of ones, the symbol ⊗ denotes the Kronecker product of matrices, and “etr” denotes the exponential of the matrix trace.

The distribution in Result 2 does not follow a known form, though it is close to the generalized Langevin-Bingham family (Hoff 2009). We sample this parameter indirectly using a parameter expansion based on the polar decomposition. The polar decomposition of the Q×K dimensional matrix X is X=UP, where $U\in R^{Q\times K}$ is orthonormal (on the Stiefel manifold ${𝒱}_{K,Q}$) and $P\in R^{K\times K}$ is positive semi-definite. The polar decomposition is unique when X is full rank K. Crucially, if X has independent N(0,1) entries, then the matrix U is uniformly distributed over the Stiefel manifold ${𝒱}_{K,Q}$ (Chikuse et al. 2003). Therefore, the uniform component of the prior on Ψ can be incorporated by imposing independent, entry-wise N(0,1) priors on X. This is a particular case of the parameter expansion strategy used by Jauch et al. (2021); Park (2024); Meng and Bouchard (2024).

FAST performs polar decomposition using the eigendecomposition $X^{t}X={ZDZ}^{t}$, defining $\Psi =X{ZD}^{-1/2}Z^{t}$ and $P=\Psi^{t}X$. It can be easily verified that $\Psi^{t}\Psi =I_{Q}$. Using this decomposition to sample Ψ works well because: (1) the element-wise independent N(0,1) priors on X are computationally simple; (2) the matrix $X\in R^{Q\times K}$ is much smaller dimensional than the matrix of eigenfunctions $\Phi \in R^{M\times K}$ when Q<<M; and (3) deterministically calculating Φ from X can be accomplished efficiently using standard linear algebra routines.

### 2.5. Alignment, Estimation, and Convergence

There are two main sources of non-identifiability for the eigenfunctions and scores in FPCA: sign flipping and ordering. This problem is exacerbated by the complex geometry of the Stiefel manifold. To address these issues, FAST incorporates both constrained MCMC sampling and post-processing steps. During sampling, strict ordering of the $\lambda_{k}$ is imposed. In post-processing, we first re-order FPCs/scores according to the score sample variances, then align the signs to maximize vector correlation between each $\phi_{k}(\cdot )$ sample and the corresponding first FPC sample after burn-in. This approach is different from existing Bayesian implementations (Goldsmith et al. 2015; Jauch et al. 2021), but it is computationally simple and performed well in our testing.

An additional problem is that the point-wise means of the FPC samples are not guaranteed to be orthonormal. To address that, we take the element-wise mean of the spline coefficient Ψ matrix and orthonormalize the results. The resulting $\hat{\Psi }$ will be orthonormal, and thus produce orthonormal FPC estimate $\hat{\Phi }=B\hat{\Psi }$ as described in Section 2.2.

To monitor convergence of FAST we use the Gelman-Rubin RHat statistic for all model parameters (Gelman and Rubin 1992). For functional components, we have RHat values at all observation points. This is done after post-processing the PCs and scores.

### 2.6. Extensions

FAST can be extended to multilevel, structured, and longitudinal functional data (Di et al. 2009; Greven et al. 2010; Shou et al. 2015), which are becoming increasingly common in applications. We demonstrate how to extend FAST to fit a multilevel model. The data structure is of the form $Y_{ij}(t),i=1,\ldots ,N,j=1,\ldots ,J_{i},t\in T=\left\{t_{1},\ldots ,t_{M}\right\}$, where there are $J_{i}$ observations for each group i. A model for such data is MFPCA (Di et al. 2009; Zipunnikov et al. 2011)

(2) $$Y_{ij}(t)=\mu (t)+\eta_{j}(t)+\sum_{k=1}^{K_{1}}\xi_{ik}\phi_{k}^{\left(1\right)}(t)+\sum_{l=1}^{K_{2}}\zeta_{ijl}\phi_{l}^{\left(2\right)}(t)+\epsilon_{ij}(t),$$

where μ(t) is the population mean and $\eta_{j}(t)$ are visit-specific deviations from the population mean. The subject-level (level 1) deviations from the visit-specific mean are modeled by $U_{i}(t)=\sum_{k=1}^{K_{1}}\xi_{ik}\phi_{k}^{\left(1\right)}(t)$, where $\phi_{k}^{\left(1\right)}(t),k=1,\ldots ,K_{1}$ are the first level eigenfunctions, $\xi_{ik}∼N(0,\lambda_{k}^{\left(1\right)})$, and $\lambda_{k}^{\left(1\right)}$ are the eigenvalues corresponding to $\phi_{k}^{\left(1\right)}(t)$. The subject/visit-specific (level 2) deviations from the subject-specific mean are modeled by $W_{ij}(t)=\sum_{l=1}^{K_{2}}\zeta_{ijl}\phi_{l}^{\left(2\right)}(t),$ where $\phi_{l}^{\left(2\right)}(t),l=1,\ldots ,K_{2}$ are the second level eigenfunctions, $\zeta_{ijl}∼N(0,\lambda_{l}^{\left(2\right)})$, and $\lambda_{l}^{\left(2\right)}$ are the eigenvalues corresponding to $\phi_{l}^{\left(2\right)}(t)$. Finally, $\epsilon_{ij}(t)∼N(0,\sigma^{2})$ is the error process. All functional components, $\epsilon_{ij}(t),U_{i}(t)$ and $W_{ij}(t)$, are assumed to be mutually independent.

The fixed effects, population mean μ(t) and the visit-specific deviations $\eta_{j}(t)$, are modeled using any penalized splines. The subject-specific $U_{i}(t)=\sum_{k=1}^{K_{1}}\xi_{ik}\phi_{k}^{\left(1\right)}(t)$ and the visit-specific $W_{ij}(t)=\sum_{l=1}^{K_{2}}\zeta_{ijl}\phi_{l}^{\left(2\right)}(t)$ can be modeled separately using the same techniques described in Sections 2.2, 2.4, and 2.5. This is the first Bayesian MFPCA implementation directly modeling the FPCs.

## 3. Bayesian Implementation in STAN

In this section, we provide the STAN implementation of FAST. STAN is a flexible probabilistic programming language which facilitates sampling from user-specified Bayesian models using Hamiltonian Monte Carlo (Carpenter et al. 2017; Betancourt 2018). We begin with the data section, which declares constants and imports the data, spline basis, and penalty matrix. We also calculate the trace of $P_{\alpha }$ in the transformed data section for the FPC smoothing parameter priors. All notation is as used in Section 2.

data {

 int N; //Number of time series

 int M; //Number of observations

 int Q; //Number of spline bases

 int K; //Number of Eigenfunctions

 matrix [N, M] Y; //Functional data

 matrix [M, Q] B; //Orthogonal spline basis

 matrix [Q, Q] P; //Penalty matrix for splines

 }

 transformed data {

 real tr_P=trace (P_alpha); //Trace of penalty

}

In the parameters block, we model the matrix X(X). Through the parameter expansion described in Section 2.4, we indirectly model Ψ using the polar decomposition of X. This block also contains the eigenvalues $\lambda_{k}$ (lambda) stored using a positive_ordered type, ordering the eigenvalues and corresponding eigenfunctions. The matrix Scores is the N×K score matrix Ξ introduced in Result 2.

parameters {

 real < lower =0 > sigma2; //Error in observation

 //Mean structure

 vector [Q] w_mu; //Population mean parameters

 real < lower =0 > h_mu; //Population mean smoothing parameter

 //Covariance structure

 positive_ordered [K] lambda; //Eigenvalues

 vector < lower =0 > [K] H; //EF smoothing parameters

 matrix [Q, K] X; //Unconstrained EF weights (X)

 matrix [N, K] Scores; //EF scores (xi)

}

The transformed parameters section contains deterministic functions of the data and model parameters. The key component of this block is the calculation of Ψ (Psi) as the orthogonal component of the polar decomposition X=ΨP. More precisely, $\Psi =XZD^{-1/2}Z^{t}$, where Z (evec_XtX) is the K×K dimensional matrix of eigenvectors of $X^{t}X$ and D (diag(eval_XtX)) is a diagonal matrix containing the corresponding K eigenvalues. Because $X\in R^{Q\times K}$ is low dimensional, all these operations are efficient.

 transformed parameters {

 //Population mean

 row_vector [M] mu = (B * w_mu)’;

 //Polar decomposition

 matrix [Q, K] Psi;

 {

 matrix [K, K] evec_XtX=eigenvectors_sym (X’* X);

 vector [K] eval_XtX=eigenvalues_sym (X’* X);

 Psi=X * evec_XtX * diag_matrix (1/sqrt

 (eval_XtX))* evec_XtX ‘;

 }

}

The model proceeds with the specification of priors within the model section. The key components are the priors on Ψ (Psi). We first include the smoothing component directly through the penalties described in Section 2.3. Then, we incorporate the uniform component by specifying independent N(0,1) priors for each entry of the latent matrix X (X). Combined with the polar decomposition in the transformed parameters block, this induces the uniform component of the prior on Ψ. FAST thus models the FPCs through the small-dimensional $X,\Psi \in R^{Q\times K}$, both constrained by the size of the spline basis and number of FPCs. This dimensionality reduction likely contributes to the much improved computational efficiency. All other priors are as described in Section 2.3.

model { //(Inverse) variance priors

 H ~ gamma (0.01, tr_P /2+0.01);

 h_mu ~ gamma (0.001, 0.001);

 lambda ~ inv_gamma (0.001, 0.001);

 sigma2 ~ inv_gamma (0.001, 0.001);

 //Mean function smoothing prior

 target += Q /2.0 * log (h_mu) - h_mu /

2.0 * w_mu ’ * P * w_mu;

 //FPC smoothing prior component

 for (i in 1: K){

 target += Q /2.0 * log (H [i]) - H [i]

/2.0 * Psi [, i]’ * P * Psi [, i];

 }

 //FPC uniform prior component

 to_vector (X) ~ normal (0, 1);

 //FPCA - based Score priors

 for (i in 1: K){

 to_vector (Scores [, k]) ~ normal (0,

sqrt (lambda [ k ]));

 } …

}

We next present the likelihood contribution. This portion of the model block computes the random deviations $\theta_{i}(t)=\sum_{k=1}^{K}\xi_{ik}\phi_{k}(t)=\sum_{k=1}^{K}\xi_{ik}\left\{B(t)\psi_{k}\right\}$. If Θ (Theta) is the N×M dimensional matrix with (i,m) entry equal to $\theta_{i}\left(t_{m}\right)$, it can be shown that $\Theta =\Xi (B\Psi {)}^{t}$. This value is computed, used, and discarded to conserve memory. The model finally incorporates the observed data contribution, following the FPCA data likelihood in Section 2.1, using a vectorized distributional statement for computational efficiency.

model { …{ //Data Likelihood

 matrix [N, M] Theta=Scores * (B*Psi)’;

//FPC linear combination

 to_vector (Y) ~ normal (to_vector

(rep_matrix (mu, N) + Theta),

 sqrt (sigma2));

 }

}

This implementation suggests how to extend FAST to more complex structures. For MFPCA, we sample two spline coefficient matrices $\Psi_{1}$ and $\Psi_{2}$. For each matrix, we independently model a latent matrix: $X_{1}$ for $\Psi_{1}$ and $X_{2}$ for $\Psi_{2}$. We assign independent N(0,1) priors to the entries of both $X_{1}$ and $X_{2}$. The $\Psi_{1}$ and $\Psi_{2}$ matrices are obtained via separate polar decompositions in transformed parameters. All other implementation components are straightforward, just requiring careful accounting of indices. We present both the implementation and simulation evaluation in Supplement Section 4.4.

## 4. Simulations

We consider the following two simulation scenarios for model (1), where $f_{k}(t)$ are taken to be the Legendre polynomials on [0, 1]:

*(S1)* Emulation of CGM data from Section 5

$$\mu (t)=140-20\times f_{2}(t),\sigma^{2}=4,\lambda_{k}=\{2250,450,150\}\;\phi_{k}(t)=\left\{f_{0}(t),\sqrt{\frac{84}{31}}\left[f_{1}(t)-0.5f_{3}(t)\right],-\sqrt{5}f_{2}(t)\right\}$$

*(S2)* Canonical FPCA example from Xiao et al. (2016)

$$\mu (t)=0,\sigma^{2}=0.35,\lambda_{k}=\{1,0.5,0.25\}\;\phi_{k}(t)=\{\sqrt{2}\;\text{sin}(2\pi t),\sqrt{2}\;\text{cos}(4\pi t),\sqrt{2}\;\text{sin}(4\pi t)\}$$

For each scenario we generate 200 datasets with N=50 functions observed at M=50 points distributed along [0, 1] according to quadrature. FAST is compared to the Generalized Function-on-Scalar Regression of Goldsmith et al. (2015) (labeled “GFSR”), modeling FPCs in the observed data space using parameter expansion based upon polar decomposition from Jauch et al. (2021) (labeled “POLAR”), and Variational Message Passing from Nolan et al. (2023) (labeled “VMP”). While all comparators fit a Bayesian FPCA, they do not all address uncertainty quantification for the same set of parameters. Neither POLAR nor VMP address construction of credible intervals for the functional components. Further, POLAR does not model the mean, instead subtracting point-wise means in pre-processing.

For the MCMC-based approaches, we performed 1500 sampling iterations and discarded the first 1000 as burn-in. This was chosen based upon the convergence properties of FAST and kept uniform between methods.

To obtain the orthonormal FPCs from the unconstrained samples of GFSR, we leverage the post-processing rotation suggested by Goldsmith et al. (2015). This involves calculating the SVD of the unconstrained FPC matrix sample and taking the right singular vectors. The scores are correspondingly transformed by the left singular vectors and diagonal matrix of singular values. This selection of a common rotation addresses the permutation- and rotation-based non-identifiability of the FPCs and scores. With a suitable alignment of sign, such as described in Section 2.5, the rotated posterior FPC/score samples can be used to form corresponding credible intervals.

Similar to FAST, both POLAR and GFSR require procedures to produce orthonormal (in the vector sense) FPC estimates. For POLAR, Jauch et al. (2021) recommends taking the right singular vectors of the mean smoothed data. As for GFSR, we take the point-wise mean of the non-orthogonal latent principal component matrix, then apply the SVD-based rotation Goldsmith et al. (2015) describes. VMP provides only FPC estimates.

POLAR, GFSR, and VMP all produce FPC estimates/samples which are orthonormal in the vector sense. So, we rescale the FPC estimates for these methods to have norm 1 in $L^{2}([0,1])$. As all methods are subject to the identifiability issues described in Section 2.5, we applied the post-processing steps described in that section to align posterior samples and estimates. FPC/score signs were then aligned with those of the true underlying FPCs for the purposes of evaluation.

**Figure 1.**
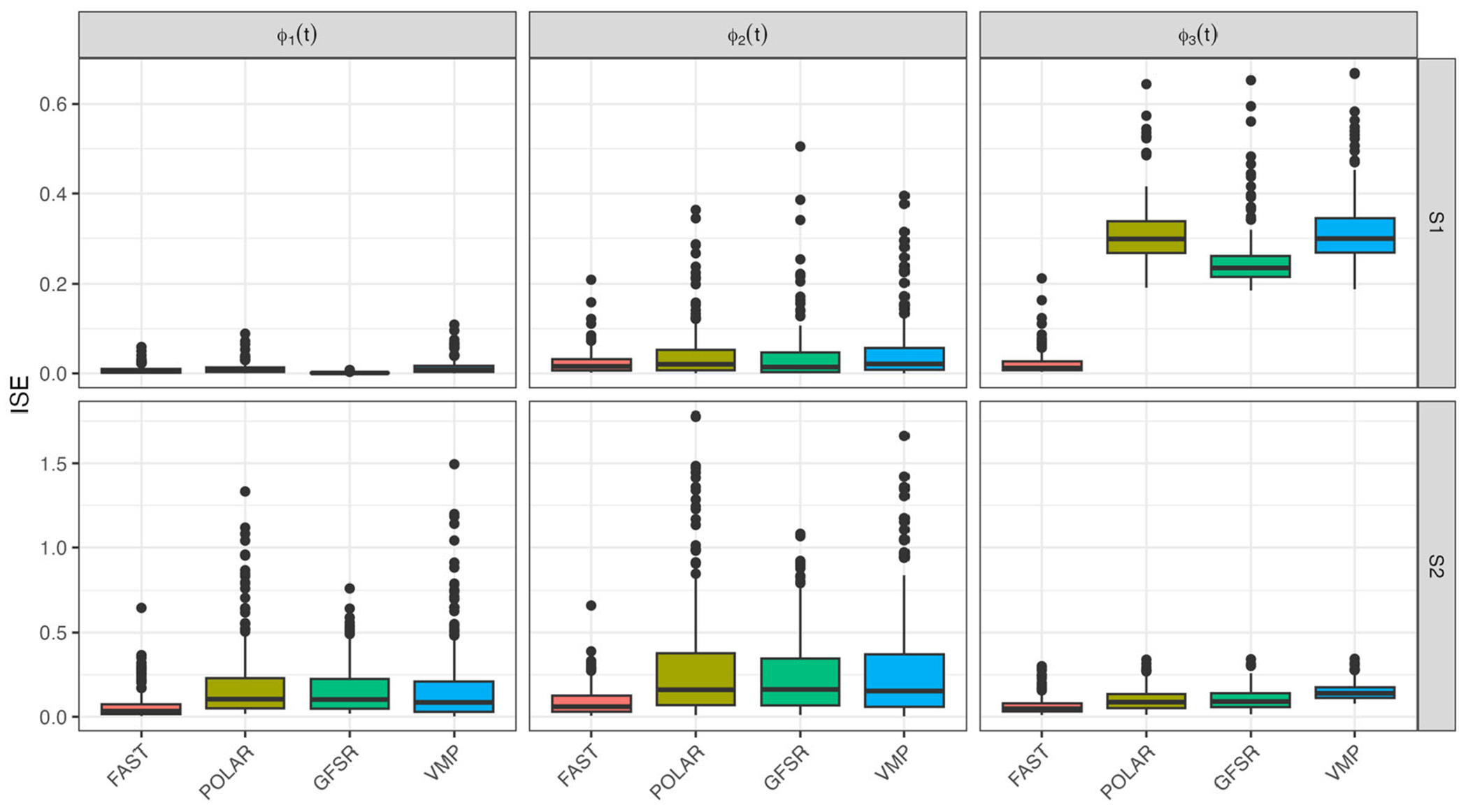
ISE boxplots for FAST, POLAR, GFSR, and VMP. Row indicates simulation scenario, S1 then S2. Each column corresponds to a particular eigenfunction.

We first focus on the Integrated Square Error (ISE) for FPCs $\phi_{k}(t)$. If ${\hat{\phi }}_{k}^{b}(t)$ is the posterior estimate of $\phi_{k}(t)$ for simulated data set b∈{1,…,B}, ISE is defined as:

$${\text{ISE}}^{b}\left\{\phi_{k}(\cdot )\right\}=\int_{0}^{1}{\left\{{\hat{\phi }}_{k}^{b}(t)-\phi_{k}(t)\right\}}^{2}dt\approx \sum_{m=1}^{M}w\left(t_{m}\right){\left\{{\hat{\phi }}_{k}^{b}\left(t_{m}\right)-\phi_{k}\left(t_{m}\right)\right\}}^{2},$$

where w(⋅) are the corresponding Gaussian quadrature weights at $t_{m},m=1,\ldots ,M$. Combining the ${\text{ISE}}^{b}$ values over simulations provides a B-dimensional vectors for each method and each principal component. Figure 1 displays the boxplots of these ISE by principal component (column) for scenario S1 (first row) and scenario S2 (second row) and method. Results indicate that FAST consistently outperforms existing methods.

We also estimate pointwise posterior coverage probabilities of the true FPCs $\phi_{k}(t),k=1,\ldots ,K$. For each simulated data set b∈{1,…,B}, we obtain the equal-tail 95% credible intervals at the observed time points $t_{m},m=1,\ldots ,M$. The proportion of times these credible intervals cover the true function is calculated over the $t_{m}$ for each combination of b and k. These proportions are then collected in a vector of length B=200 for each function $\phi_{k}(t)$. VMP and POLAR are excluded from this comparison, as they do not address uncertainty quantification for the FPCs.

**Figure 2.**
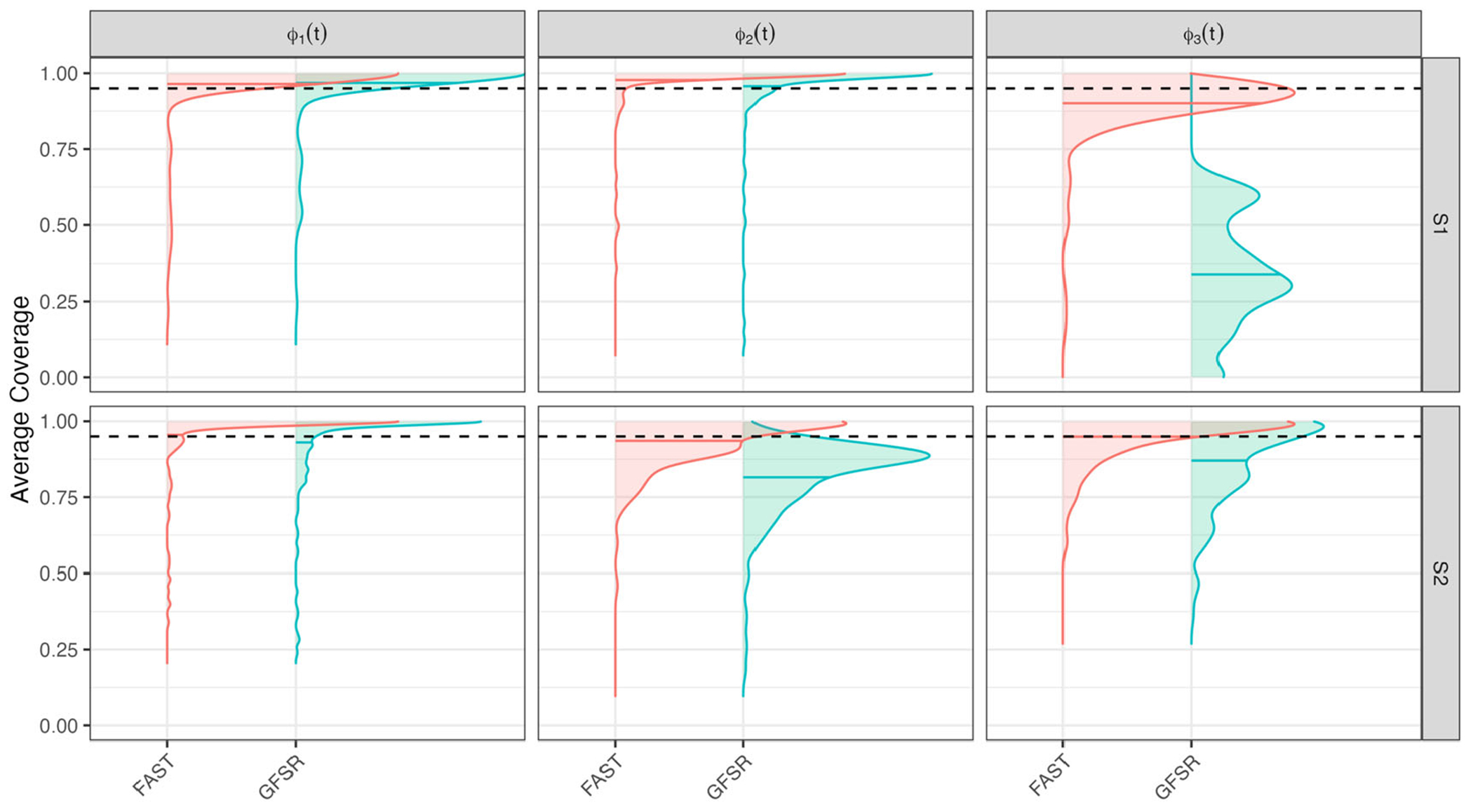
Kernel smoother of 95% credible interval coverage probabilities of the true FPCs for FAST, POLAR, and GFSR. Row indicates simulation scenario, S1 then S2. Each column corresponds to a particular eigenfunction. Distribution means: horizontal solid lines; nominal 95% level: horizontal dotted lines.

Figure 2 displays the kernel smooth of the estimated pointwise coverage proportions for FAST and GFSR using the same structure as Figure 1. FAST performs well in all scenarios, consistently out-performing GFSR.

Figures 1 and 2 indicate that the advantage of FAST is particularly striking for $\phi_{3}(t)$ in S1. In this case, the mean can be written as a linear combination of $\phi_{1}(t)$ and $\phi_{3}\left(t\right):\mu (t)=140\phi_{1}(t)+4\sqrt{5}\phi_{3}(t)$. This choice makes the problem more challenging in finite samples, when information may leak between the mean and covariance; see, for example, Zhou et al. (2025) for a more detailed discussion.

For inference on the mean, μ(t), we only compared to GFSR, as neither the VMP nor POLAR perform inference on this component. Results indicate that FAST produces smaller ISE and better coverage probability; see Supplement Section 4.2 for more details.

We also compared estimation accuracy and posterior coverage of credible intervals for the scores $\xi_{k}$. Accuracy is calculated as mean square error aggregated by FPC, k, within each simulation, b. Coverage was calculated as the proportion of times the equal-tail 95% credible intervals cover the corresponding true scores; see results in Supplement Section 4.3. FAST outperforms the other methods and has near nominal coverage.

Table 1 provides computation times in minutes on a personal laptop (MacBook Pro, 3.49 GHz and 32 GB RAM) as both N (number of subjects) and M (number of observation points) scale up for scenario S1. The left column provides times for N=50 and M∈{50,100,250,500}, while the right provides times for M=50 and N∈{50,100,250,500}.

FAST and GFSR scale approximately linearly with M, though GFSR has a substantially higher slope. FAST and POLAR scale linearly in N, while GFSR seems to be more performant with higher N, possibly due to the increased information better specifying the posterior geometry. We could not fit POLAR for M≥250 because it uses too much memory. VMP is the fastest, which is not unexpected given the approximation inherent to the technique. All MCMC-based methods were run for 1500 iterations with the first 1000 discarded as burn-in; however, the convergence characteristics were not uniform. FAST and POLAR exhibit lower RHats (≤ 1.05) compared to GFSR (≤ 1.25), which may require a longer warm-up. The computation time of FAST is relatively robust to different Q and K values (see Supplement Section 5.4).

Simulations for the multilevel scenario provide similar results; see Supplement Section 4.4. POLAR was not included because it does not have a multilevel implementation.

## 5. Data Analysis: The DASH4D CGM Study

Dietary Approaches to Stop Hypertension for Diabetes (DASH4D, NCT04286555) is a nutritional trial designed to assess how blood pressure and glucose respond to combinations of DASH4D-style and low-sodium diets in persons with type 2 diabetes (T2D). The study team prepared four diets: DASH4D diet with lower sodium, DASH4D diet with higher sodium, Comparison diet with lower sodium, and Comparison diet with higher sodium. Weight was held constant by adjusting calorie level. Diet effects were observed through a single-site, 4-period crossover design (Pilla et al. 2025). Each period consisted of 5 weeks of feeding a diet followed by a ≥ 1-week break (median 2 weeks). Approval for DASH4D was granted by the Johns Hopkins University School of Medicine institutional review board, and all study participants provided informed written consent. There were N=105 randomized T2D participants recruited from the Baltimore area, of which N=65 had meal timing data. Participants with meal timing data had a median age of 68 years, were 66% female, and had the following race distribution: 6.2% Asian, 87.7% Black, and 6.2% White.

The DASH4D CGM sub-study was conducted to evaluate the impact of the dietary intervention on glucose assessed by CGM. Participants wore the Abbott Freestyle Libre Pro (Abbott Diabetes Care), placed near the middle of the third week and worn into the fifth week for each feeding period. The CGM devices were placed on the back of the upper arm (approved location) by trained technicians. These devices record interstitial glucose every 15 minutes. The Libre Pro is a masked CGM system, so participants were not aware of the glucose measurements (Wang et al. 2025).

### 5.1. Mealtime CGM Glucose

During feeding periods, participants ate meals at the study site 3 days each week. Clinic staff observed consenting participants to document meal timing. We used these timestamps to extract CGM data starting 1 hour before and ending 4 hours after each meal began. There were ≤ 20 such 5– hour periods per participant. The final dataset was comprised of 768 meals over 65 individuals. This data is not yet available as trial results are forthcoming.

**Figure 3.**
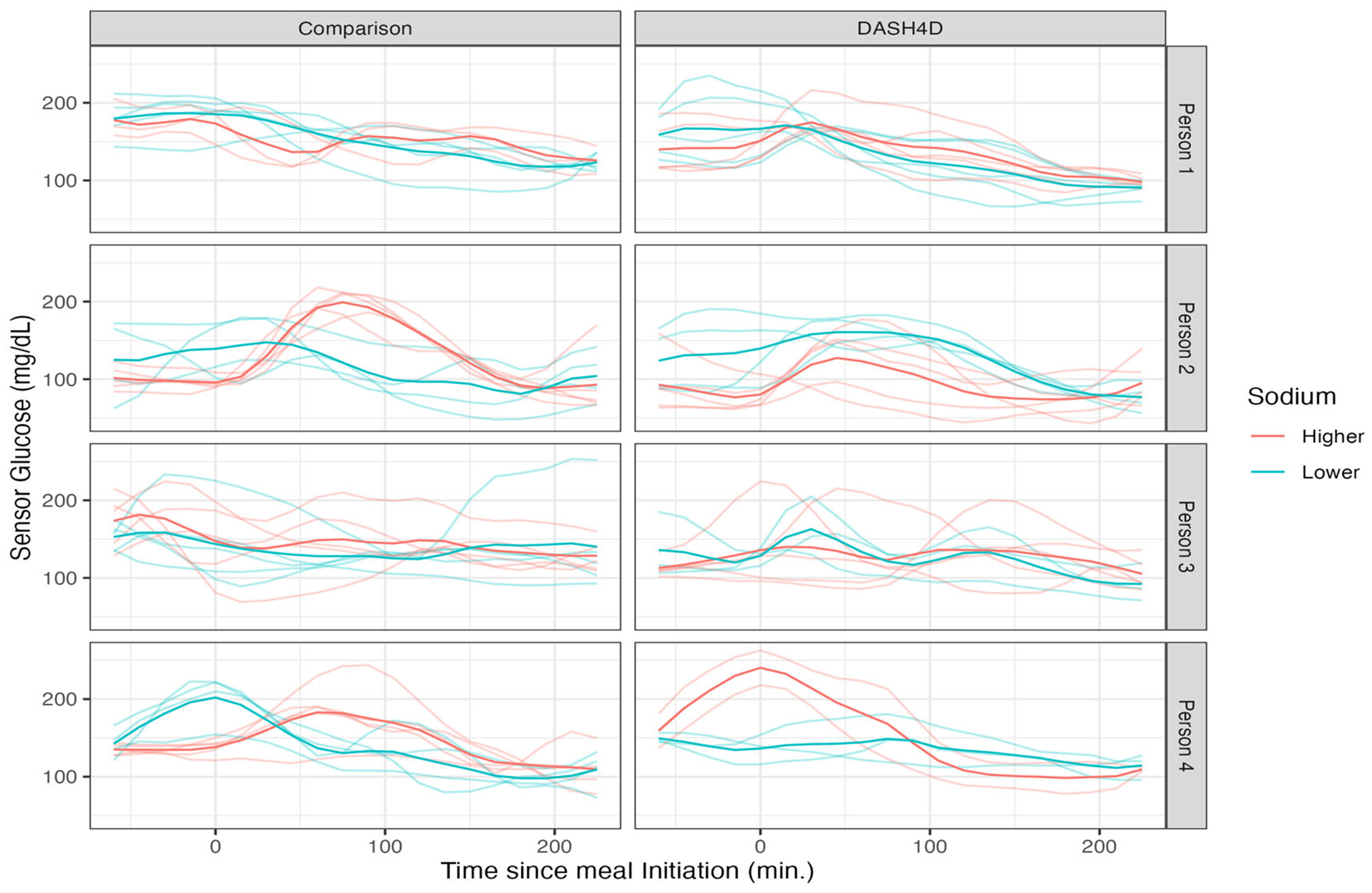
Mealtime CGM for 4 participants (one per row), for the comparison (left column) and DASH4D (right column) diets. Sodium level within diet type is indicated by color. X-axis: time from meal initiation, y-axis: CGM in mg/dL. Person- and diet-specific average curves are darker, while meal-specific curves are lighter.

Figure 3 provides a visualization of mealtime CGM for four randomly-sampled study participants. The x-axis in each panel corresponds to time in minutes from meal initiation; the y-axis is the recorded glucose. Each row corresponds to a participant. The first column is for Comparison meals, and the second is for DASH4D meals. Curve color corresponds to sodium level, and darker curves are within-person averages. Given this data structure, one could model the average curves within person using a single-level FPCA or the meal-specific trajectories using a two-level FPCA to capture both the participant-specific averages (level 1) and the meal-specific deviations (level 2) within each diet. We undertake these analyses in Sections 5.2 and 5.3, respectively.

### 5.2. Bayesian FPCA of Average CGM within Diets

We fit FAST to the participant average CGM curves for each diet separately (darker curves in Figure 3) using a spline basis of dimension Q=20 and K=3 FPCs. This number of FPCs explained ≥ 95% of the variability across diets (see Supplemental Figure S15) The number of curves per-diet ranges between 36 and 49, so accounting for variability in FPC estimates is of particular importance (Goldsmith et al. 2013).

**Figure 4.**
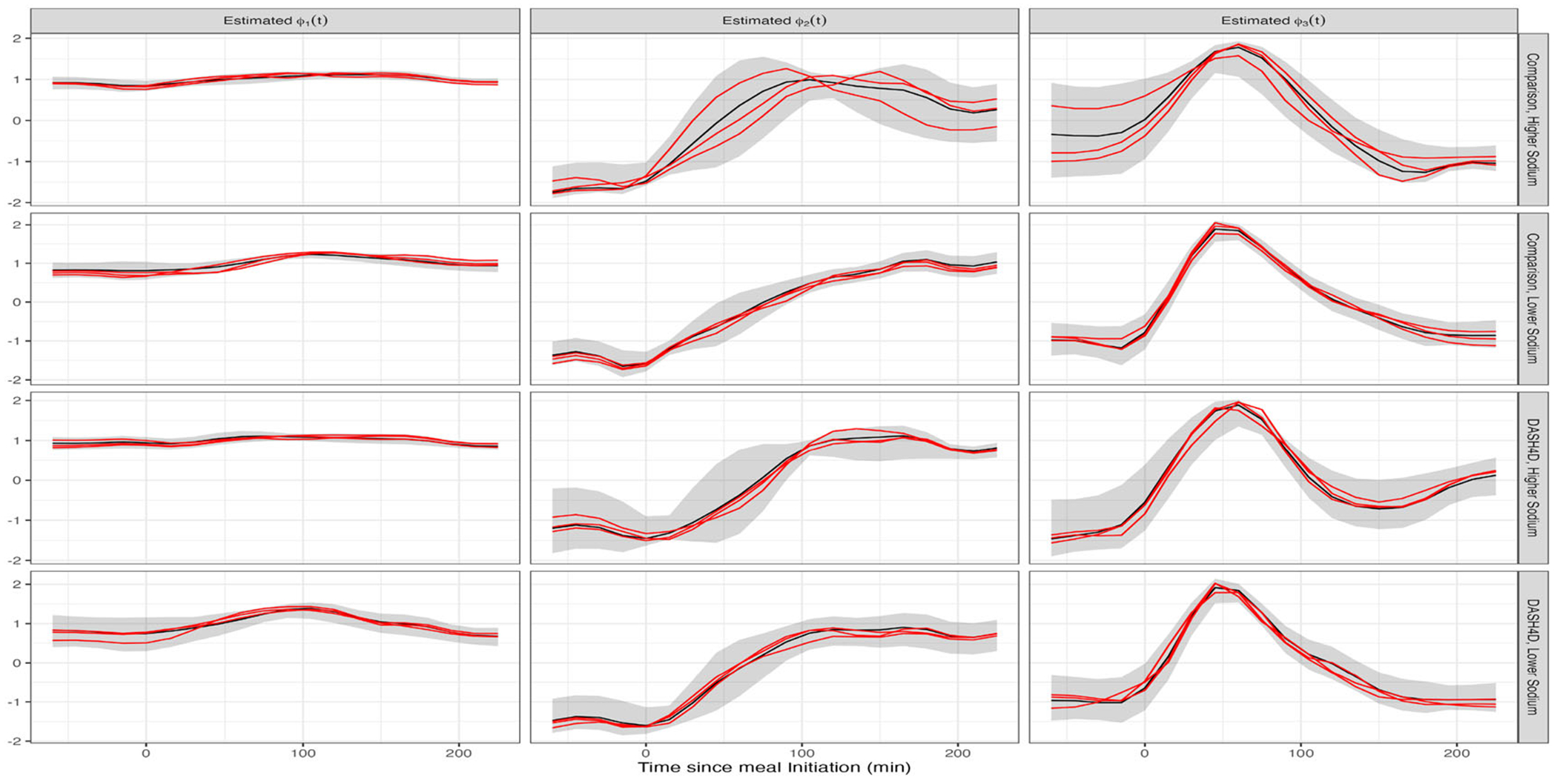
Bayesian FPCA results for the first three FPCs (columns) for each of the four diets (rows). X-axis: time relative to meal initiation. Black curves: posterior mean; red curves: three FPC posterior samples; shaded areas: point-wise 95% credible intervals.

Figure 4 displays results for Bayesian FPCA inference for the first three PCs (columns) for the four diets (rows). For all panels the x-axis is the time from the start of the meal, the black curves are the posterior means, the red curves are three samples from the posterior of the PCs, and the shaded areas are point-wise, equal-tail 95% credible intervals. The red curves correspond to the same sample iterations across PCs.

**Figure 5.**
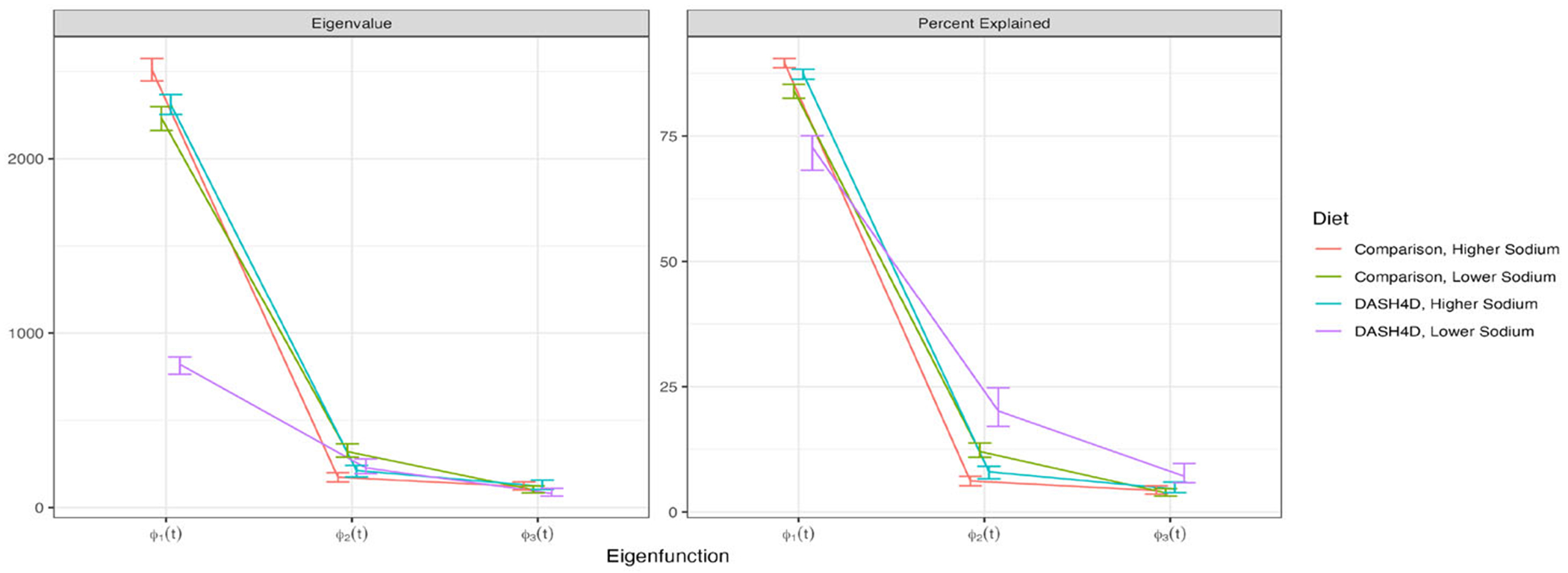
Eigenvalues (left panel) and percent-variability (right panel) estimates and 95% credible intervals for each of the four diets (one color corresponds to one diet).

Figure 5 provides the eigenvalues (left panel) and percent variance explained (right panel) for the eigenfunctions displayed in Figure 4. Diet is indicated by color.

Posterior eigenfunction estimates ${\hat{\phi }}_{k}(t)$ are qualitatively similar across diets in Figure 4. The primary modes of variability ${\hat{\phi }}_{1}(t)$ are nearly constant with a small curvature. Higher scores on these components correspond to higher CGM responses over the entire mealtime period. The sample-to-sample variability in the shape of $\phi_{1}(t)$ is low. Moreover, $\phi_{1}(t)$ explains 70 to 85% of the observed variability. This quantifies the importance of the mean postprandial glucose level (Garber 2012). The second FPCs, ${\hat{\phi }}_{2}(t)$, generally exhibit an S pattern. Therefore, individuals with a higher positive score on ${\hat{\phi }}_{2}(t)$ have lower glucose in the pre-prandial and immediate postprandial period with a larger response one to four hours after eating. The third FPCs, ${\hat{\phi }}_{3}(t)$, are negative during the hour before the meal, increase to a peak positive value about 50 to 70 minutes after food intake, and decrease back to negative values about 150−200 minutes after the meal. Therefore, individuals with higher scores on ${\hat{\phi }}_{3}(t)$ tend to have a to lower glucose before the meal and a rapid glucose increase after the meal. Sample-to-sample variability of both the $\phi_{2}(t)$ and $\phi_{3}(t)$ is higher than that of $\phi_{1}(t)$, likely due to the smaller signal.

The variability of glucose response in the DASH4D/lower sodium diet is much lower than for the other three diets. Figure 5 indicates that ${\hat{\lambda }}_{1}(t)$ is ≈3 times smaller for this diet compared to the others. This diet, a primary interest of the DASH4D study, appears to be associated with a more stable glycemic response between participants.

FAST produces reasonable results, qualitatively consistent with standard FPCA, across all four diets. Each model takes 10−40 seconds (depending on the diet) to fit on a personal laptop using 2000 iterations with the first 1000 discarded as burn-in. We employ the routine from Section 2.5 to assess convergence of the scores, $\xi_{ik}$, and sampled points along the domain for each FPC, $\phi_{k}\left(t_{m}\right)$. Final Gelman-Rubin statistics were < 1.05. Despite differences in number of meals per participant, results were similar when fitting FPCA to randomly sampled meals; see Supplement Section 6.2.

### 5.3. Bayesian MFPCA of Individual CGM Curves

So far, we have ignored the variability of meal-specific CGM curves around the subject-specific mean. This could raise questions about accounting for all observed variability, different number of meals per person, and representativeness of the average curves. To address these problems, we fit the multilevel extension of FAST described in Section 2.6 for each diet separately, with meals nested within participants. We use a rich spline basis of dimension $Q=20,K_{1}=2$ FPCs at the subject level, and $K_{2}=3$ FPCs at the meal level. These choices explain ≥ 80% variability at both levels. The number of observed meals ranges from 147 to 219 for each diet, with at most five meals per participant. There is no reason to assume that meal/visit order has effect, so we set $\eta_{j}(t)=0$ for all j.

**Figure 6.**
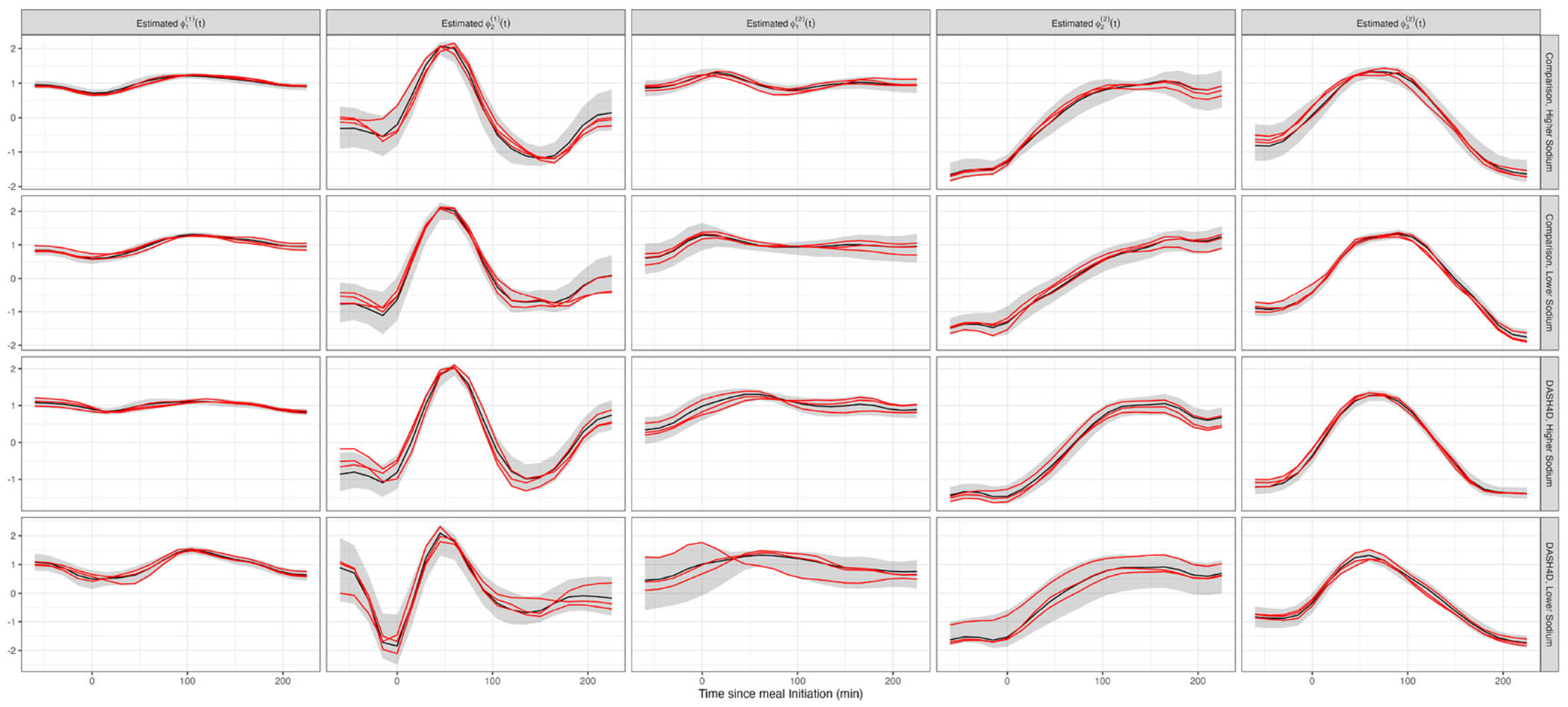
Bayesian MFPCA results for the first two PCs at the subject level and first three PCs at the meal level. Each column corresponds to an FPC and each row corresponds to a diet. X-axis: time from the meal start. Plotting conventions are consistent with Figure 4.

Figure 6 displays the Bayesian MFPCA results, with row indicating diet and column indicating FPC. The first two columns correspond to the subject level, while the last three correspond to the meal level. Plotting conventions are consistent with those in Figure 4.

**Figure 7.**
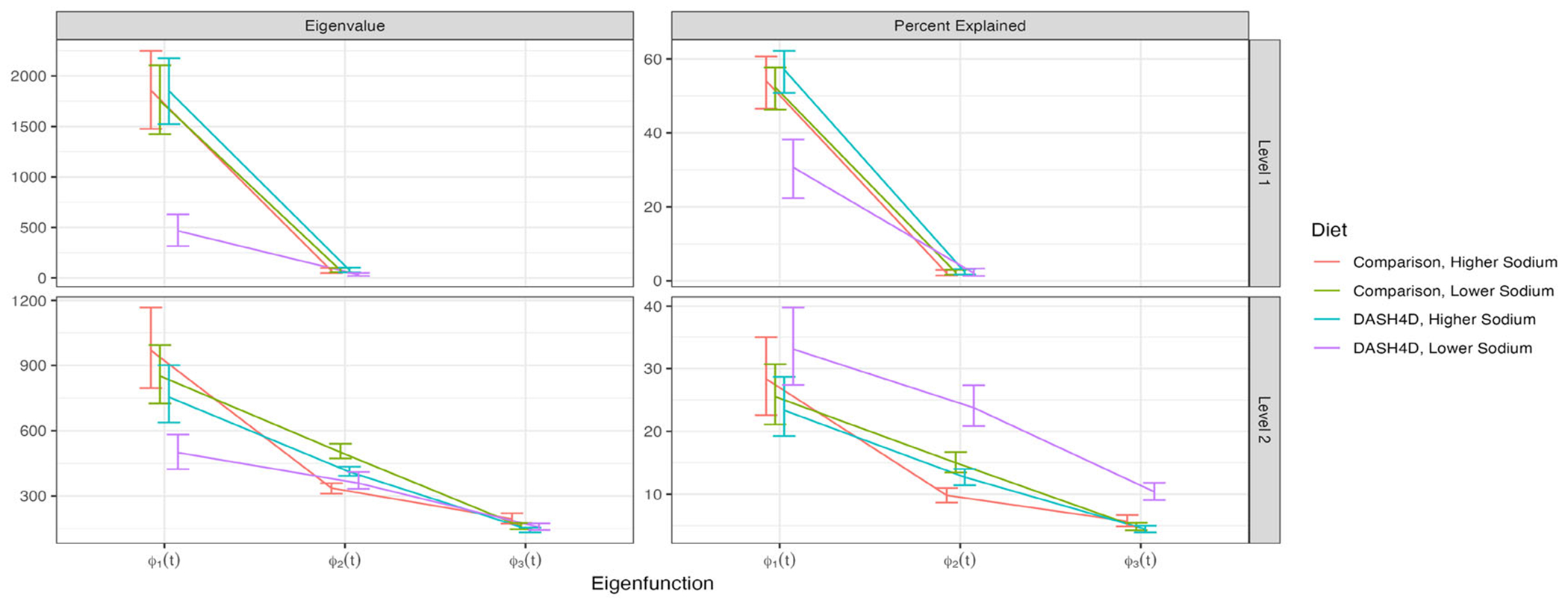
Eigenvalues (left panels), percent-variability explained (right panels) within level (first row: level 1; second row: level 2) and diet (each diet shown in one color). All plotting conventions are consistent with Figure 5

Figure 7 provides the estimated variability (left panels) and percent variability explained (right panels) within level and diet. The first row of panels corresponds to the subject (level 1) while the second row of panels corresponds to the meal (level 2). Each color corresponds to a diet. As in the single level analyses, Figure 6 indicates qualitative similarity of estimates ${\hat{\phi }}_{k}^{\left(1\right)}(t),{\hat{\phi }}_{l}^{\left(2\right)}(t)$ across diets. The primary mode of variability within each diet is ${\hat{\phi }}_{1}^{\left(1\right)}(t)$, a nearly constant function. Higher subject scores on this component correspond to higher subject-specific glucose over the entire observation window. The sample-to-sample variability in the shape of $\phi_{1}^{\left(1\right)}(t)$ is consistently low, indicating confidence in this functional form. There is some heterogeneity in the percent variability explained by this component, but it is ≥ 30% for every diet. The first eigenfunction at the second level, ${\hat{\phi }}_{1}^{\left(2\right)}(t)$, is also nearly constant with a small curvature and low sampling variability. This FPC explains between 25 – 35% of the total variability according to Figure 7, indicating that a constant shift is the main source of variation in glucose responses both among and within participants. The second eigenfunction at the meal level, ${\hat{\phi }}_{2}^{\left(2\right)}(t)$, explains 10 – 25% of the total variability and has an S pattern. As in the single-level case, larger scores on ${\hat{\phi }}_{2}^{\left(2\right)}(t)$ correspond to lower pre- and immediately post-prandial glucose, then increasingly larger response one to four hours after the meal. The eigenfunctions ${\hat{\phi }}_{2}^{\left(1\right)}(t),{\hat{\phi }}_{3}^{\left(2\right)}(t)$ capture higher-order glycemic reaction patterns and explain much smaller proportions of variability (≤ 10%).

As in the single level case, the DASH4D/lower sodium diet exhibits less variability among subject-specific glucose response (compare first row, first column in Figure 7 to the first panel in Figure 5). This diet also has lower meal-to-meal variability, evident in the second row, first column of Figure 7. Across all diets, the second eigenvalues at the subject level are very small, particularly compared to the first two eigenvalues at the meal level (compare the first row, first column and second row, first column panels in Figure 7). This suggests that most variability can be partitioned in a constant vertical shift at the subject level and more complex meal-to-meal variation.

Each FAST MFPCA model takes 1 – 3 minutes to run 2000 iterations on a standard laptop, with the first 1000 discarded as burn-in. We again use the routine described in Section 2.5 to assess convergence of the scores and FPCs at both levels. This requires aligning each level separately. Final Gelman-Rubin statistics were < 1.05.

## 6. Discussion

Standard implementations of FPCA use a two-step procedure: (1) diagonalize a smooth estimate of the observed data covariance; and (2) conduct inference conditional on the FPC estimates from the first step. This does not account for sampling variability in the FPCs, which could be quite large for low to moderate signal-to-noise. Our approach is a single-step joint Bayesian model that treats PCs as parameters in the model, which combines the orthogonal spline expansion from Ye (2024), the parameter expansion based on polar decomposition from Jauch et al. (2021), and stabilizing constraints to efficiently jointly model all FPCA model components. Combining the right ingredients led to FAST, a stable, replicable, and easy to implement methodology. The central element of FAST is expressing the FPCs in terms of an orthonormal spline basis. This is different from the approach in Ye (2024), as this spline basis: (1) is orthonormal in $L^{2}([0,1])$, rather than in a vector sense; and (2) retains the computational efficiency of B-splines (Liu et al. 2020). This spline expansion simplifies the problem of sampling the high dimensional FPCs to sampling a low dimensional spline coefficients. The FPC spline coefficient matrix must be orthonormal to produce the corresponding FPC samples. This is accomplished using the polar decomposition parameter expansion of Jauch et al. (2021), applying the technique to the coefficient matrix rather than the data level FPCs.

FAST performs as well or better than all existing methods, while being highly computational efficient and having excellent mixing properties. Moreover, the implementation of FAST within STAN, or equivalent software, allows for a widespread use of methods and requires only small adjustments for when tailoring the model for other applications.

## Supplementary Material

Supp 1

Supplementary materials for this article are available online. Please go to www.tandfonline.com/r/JCGS.

## Figures and Tables

**Table 1.** Computation times (in minutes) for each combination of scale and method (simulation S1).

Fixed *N* = 50					Fixed *M* = 50				
*M*	FAST	GFSR	POLAR	VMP	*N*	FAST	GFSR	POLAR	VMP
50	3.5	24.3	9.2	0.2	50	3.5	24.3	9.2	0.2
100	6.8	39.5	28.5	1.0	100	4.3	43.2	15.4	0.6
250	18.3	82.8	—	1.2	250	5.3	47.2	29.0	0.6
500	37.6	151.0	—	5.2	500	14.7	27.8	52.1	0.7

All methods evaluated on the same simulated dataset for each *N, M* combination.
