# Supplementary material for: Fast Bayesian Functional Principal Components Analysis
Source: J Comput Graph Stat. Author manuscript; Available in PMC 2026 Apr 7. (PMC13053144; doi:10.1080/10618600.2025.2592768)
Supplement: Supp 1 [file NIHMS2149685-supplement-Supp_1.pdf]

SUPPLEMENTARY MATERIAL

1 Penalty matrix $\mathbf{P}_\alpha$

Recall we chose a posterior addition which mixes second- and zero-order penalties of the form $\alpha \int f^2(t)dt + (1-\alpha)\int \{f''(t)\}^2 dt$. For spline parameters θ such that $f(t) \approx \mathbf{B}(t)\theta$, there exist unique penalty matrices $\mathbf{P}_0, \mathbf{P}_2$ such that $\int f^2(t)dt \approx \theta^t \mathbf{P}_0 \theta$ and $\int \{f''(t)\}^2 dt \approx \theta^t \mathbf{P}_2 \theta$ (Craven and Wahba, 1979; Kimeldorf and Wahba, 1970; O'Sullivan, 1986; Wahba, 1983).

We proceed by defining both of the penalty components $\mathbf{P}_0, \mathbf{P}_2$ separately. Let $\mathbf{B}(t) = [b_1(t)|\ldots,|b_Q(t)]$ represent the basis functions, chosen to be orthonormal in $L^2([0,1])$. First, we define the zero-order penalty $\mathbf{P}_0$ element-wise. This derivation leverages the orthonormal definition of the $b_i(t)$.

$$
\begin{aligned}
(\mathbf{P}_0)_{ij} &= \int_0^1 \int_0^1 b_i(t) b_j(t) dt \\
&= \begin{cases} 1 \text{ when } i = j \\ 0 \text{ otherwise} \end{cases}
\end{aligned}
$$

The resulting matrix $\mathbf{P}_0 = \mathbf{I}_Q$ by the definition of the basis $\mathbf{B}(t)$.

Next, we define the more central "wiggliness" penalty $\mathbf{P}_2$. For this penalty, based on the squared second derivative, we introduce the second derivatives of the basis $\mathbf{B}(T)$: $\mathbf{B}''(t) = [b_1''(t)|\ldots,|b_Q''(t)]$. We are able to quickly retrieve these derivatives using the properties of B-splines, from which the default Splinet basis is constructed (Liu et al., 2020). The elements of $\mathbf{P}_2$ are as follows.

$$
(\mathbf{P}_2)_{ij} = \int_0^1 \int_0^1 b_i''(t) b_j''(t) dt
$$

We approximate these quantities using numerical integration.

To ensure uniformity of penalization scale between $\mathbf{P}_0$ and $\mathbf{P}_2$, we scale both raw penalties such that their leading eigenvalues are 1. We then follow Goldsmith et al. (2015) and define $\mathbf{P}_\alpha = \alpha\mathbf{P}_0 + (1-\alpha)\mathbf{P}_2$. This allows us to write the final penalty using a single quadratic form:

$$\begin{aligned}\alpha\theta^t\mathbf{P}_0\theta + (1-\alpha)\theta^t\mathbf{P}_2\theta &= \theta^t(\alpha\mathbf{P}_0 + (1-\alpha)\mathbf{P}_2)\theta \\ &= \theta^t\mathbf{P}_\alpha\theta\end{aligned}$$

Considering the form of $\mathbf{P}_\alpha$, it becomes clear that $\mathbf{P}_0$ acts to ensure the non-singularity of the final penalty $\mathbf{P}_\alpha$ when $\alpha > 0$, similar to adding a ridge penalty in the context of regression.

2 Joint Prior on h_k and $\mathbf{\Psi}$

We derive sufficient conditions under which the joint prior distribution on the smoothing parameters h_k and the FPC spline coefficient matrix $\mathbf{\Psi}$ is proper. This is equivalent to showing under what assumptions it can be integrated to a constant, finite value. We assume here that $\mathrm{R}(\mathbf{P}_\alpha) = Q$, as this is true for the tested implementation. Letting K refer to the dimension of the FPC basis, $\Gamma(x|a,b)$ refer to a Gamma distribution with expectation a/b evaluated at x, $\mathbf{H} = \mathrm{diag}(h_1, \ldots, h_K)$, and $\mathrm{etr}(\cdot)$ refer to the exponential of the matrix trace, we proceed:

$$\begin{aligned}g(\mathbf{\Psi}, \mathbf{H}) &= g_\psi(\mathbf{\Psi}|\mathbf{H})g_h(\mathbf{H}) \\ &\propto \frac{\mathrm{etr}(-\mathbf{H}\mathbf{\Psi}^T\mathbf{P}_\alpha\mathbf{\Psi}/2)}{\mathrm{Vol}(\mathcal{V}_{K,Q})} \times \prod_{i=1}^{K} h_i^{Q/2}\Gamma(h_i|\alpha_\psi, \beta_\psi)\end{aligned}$$

With the form of the density defined, we now integrate over the Stiefel manifold $\mathbf{\Psi} \in \mathcal{V}_{K,Q}$ and the Gamma-distributed smoothing parameters $h_k \geq 0$. Here, we use that $g_\psi(\mathbf{\Psi}|\mathbf{H})$ is proportional to a Matrix Bingham distribution with arguments $\mathbf{H}$ and $-\mathbf{P}_\alpha/2$ (using commutativity of scalar and matrix multiplication). This distribution has normalizing constant $\Phi_{K,Q}(\mathbf{H}, -\mathbf{P}_\alpha/2)^{-1}$, where Φ is the hypergeometric function of two matrix arguments. This function has the following series definition:

$$\Phi_{K,Q}(\mathbf{H}, -\mathbf{P}_\alpha/2) = \sum_{k=0}^{\infty} \frac{1}{k!} \sum_{|\kappa|=k} \frac{C_\kappa(\mathbf{H})C_\kappa(-\mathbf{P}_\alpha/2)}{C(I_K)}$$

where $C_\kappa()$ denotes the zonal polynomial related to partition κ and the inner sum is over all partitions κ of weight k and length $\leq K$. Using this normalizing constant, we can integrate the joint prior density of the smoothing parameters and FPC spline coefficients as follows, letting $V = \text{Vol}(\mathcal{V}_{K,Q})$. Differentials are omitted for notational brevity.

$$\begin{aligned}
\int_0^\infty \cdots \int_0^\infty \int_{\boldsymbol{\Psi} \in \mathcal{V}_{K,Q}} g(\boldsymbol{\Psi}, \mathbf{H}) &= \int_0^\infty \cdots \int_0^\infty \int_{\boldsymbol{\Psi} \in \mathcal{V}_{K,Q}} \frac{\text{etr}(-\mathbf{H}\boldsymbol{\Psi}^T \mathbf{P}_\alpha \boldsymbol{\Psi}/2)}{V} \times \prod_{i=1}^K h_i^{Q/2} \Gamma(h_i | \alpha_\psi, \beta_\psi) \\
&= \frac{1}{V} \int_0^\infty \cdots \int_0^\infty \Phi_{K,Q}(\mathbf{H}, -\mathbf{P}_\alpha/2) \times \prod_{i=1}^K h_i^{Q/2} \Gamma(h_i | \alpha_\psi, \beta_\psi) \\
&= \frac{1}{V} \int_0^\infty \cdots \int_0^\infty \sum_{k=0}^\infty \frac{1}{k!} \sum_{|\kappa|=k} \frac{C_\kappa(\mathbf{H}) C_\kappa(-\mathbf{P}_\alpha/2)}{C_\kappa(I_K)} \times \prod_{i=1}^K h_i^{Q/2} \Gamma(h_i | \alpha_\psi, \beta_\psi)
\end{aligned}$$

At this point, we recall that the zonal polynomials are homogeneous (order equal to the weight $|\kappa|$), symmetric polynomials in the eigenvalues of the argument matrix. For positive definite matrix $\mathbf{P}_\alpha$, the eigenvalues of $-\mathbf{P}_\alpha/2$ will be negative. The negative sign contribution from each such eigenvalue will be be present in every factor within each term of the polynomial, with cumulative power corresponding to the uniform order $|\kappa|$. This implies that $C_\kappa(-\mathbf{P}_\alpha/2) = (-1)^{|\kappa|} C_\kappa(\mathbf{P}_\alpha/2)$. Using this fact, the integral of interest becomes the following.

$$\int_0^\infty \cdots \int_0^\infty \int_{\boldsymbol{\Psi} \in \mathcal{V}_{K,Q}} g(\boldsymbol{\Psi}, \mathbf{H}) = \frac{1}{V} \int_0^\infty \cdots \int_0^\infty \sum_{k=0}^\infty \frac{(-1)^k}{k!} \sum_{|\kappa|=k} \frac{C_\kappa(\mathbf{H}) C_\kappa(\mathbf{P}_\alpha/2)}{C_\kappa(I_K)} \times \prod_{i=1}^K h_i^{Q/2} \Gamma(h_i | \alpha_\psi, \beta_\psi)$$

We now recall the combinatorial definition of zonal polynomials. In particular, each zonal polynomial $C_\kappa(A)$ for partition κ and matrix $A \in m \times m$ can be represented as a linear combination of the monomial symmetric functions $M_\lambda(\cdot)$ in the eigenvalues of A $(\sigma_1 \ldots, \sigma_m)$:

$$C_\kappa(A) = \sum_{\lambda \leq \kappa} c_{\kappa,\lambda} M_\lambda(\sigma_1, \ldots, \sigma_m)$$

These monomial symmetric functions $M_\lambda(\cdot)$ correspond to partitions λ which have equal weight to $(|\lambda| = |\kappa|)$ and are before κ according to lexicographic ordering. All coefficients $c_{\kappa,\lambda}$ are non-negative. The monomial symmetric functions are defined as follows for partition λ of length $\mathcal{L}$.

$$M_\lambda(\sigma_1, \ldots, \sigma_m) = \sum_{p \in P} \sigma_{p_1}^{\lambda_1} \cdots \sigma_{p_\mathcal{L}}^{\lambda_\mathcal{L}}$$

where the summation is taken over all distinct permutations $p \in P$ of $\mathcal{L}$ elements drawn from $\{1, \ldots, m\}$ (Bagyan and Richards, 2024). By this definition, it follows that the zonal polynomial of a positive semi-definite matrix will be positive when the length of the partition is at most the rank of the matrix, taking zero-value when the length is greater. Note that the summation over partitions κ above requires that all have length $\leq K < Q$.

As $\mathbf{P}_\alpha/2$ and I_K are positive definite, it follows that $C_\kappa(\mathbf{P}_\alpha/2) > 0$ and $C_\kappa(I_K) > 0$. Similarly, $\mathbf{H}$ being positive semi-definite implies that $C_\kappa(\mathbf{H}) \geq 0$. This, combined with the non-negativity of each factor $\frac{h_i^{Q/2}}{k!}\Gamma(h_i|\alpha_\psi, \beta_\psi)$, implies that the sign of the series indexed by k is entirely decided by the $(-1)^k$ term. Using this fact, we can break the summation into positive and negative portions.

$$\int_0^\infty \cdots \int_0^\infty \int_{\mathbf{\Psi} \in \mathcal{V}_{K,Q}} g(\mathbf{\Psi}, \mathbf{H}) = \frac{1}{V} \int_0^\infty \cdots \int_0^\infty \sum_{k=0}^\infty \frac{1}{(2k)!} \sum_{|\kappa|=2k} \frac{C_\kappa(\mathbf{H}) C_\kappa(\mathbf{P}_\alpha/2)}{C_\kappa(I_K)} \times \prod_{i=1}^K h_i^{Q/2} \Gamma(h_i|\alpha_\psi, \beta_\psi)$$
$$- \frac{1}{V} \int_0^\infty \cdots \int_0^\infty \sum_{k=0}^\infty \frac{1}{(2k+1)!} \sum_{|\kappa|=2k+1} \frac{C_\kappa(\mathbf{H}) C_\kappa(\mathbf{P}_\alpha/2)}{C_\kappa(I_K)} \times \prod_{i=1}^K h_i^{Q/2} \Gamma(h_i|\alpha_\psi, \beta_\psi)$$

We will now apply Tonelli's theorem to each part, once for each Gamma-distributed smoothing parameter. To verify that Tonelli's theorem applies, we consider the necessary assumptions: (1) the measure spaces integrated over must be $\sigma-$finite, (2) the integrand must be non-negative and, (3) the integrand must be measurable. Each application of Tonelli's theorem here applies to a function $f(h_j, k)$ as defined for the first and second terms below. We use here that zonal polynomials are polynomial functions of the eigenvalues, which are just the diagonal elements for a diagonal matrix such as $\mathbf{H}$.

$$\text{Term 1: } f(h_j, k) = \frac{1}{(2k)!} \sum_{|\kappa|=2k} \frac{C_\kappa(h_1, \ldots, h_j, \ldots, h_K) C_\kappa(\mathbf{P}_\alpha/2)}{C_\kappa(I_K)} \times \prod_{i=1}^K h_i^{Q/2} \Gamma(h_i|\alpha_\psi, \beta_\psi)$$
$$\text{Term 2: } f(h_j, k) = \frac{1}{(2k+1)!} \sum_{|\kappa|=2k+1} \frac{C_\kappa(h_1, \ldots, h_j, \ldots, h_K) C_\kappa(\mathbf{P}_\alpha/2)}{C_\kappa(I_K)} \times \prod_{i=1}^K h_i^{Q/2} \Gamma(h_i|\alpha_\psi, \beta_\psi)$$

We consider the $f(h_j, k)$ for both terms concurrently. In these functions, $h_j \in (\mathbb{R}^+, \mathbf{B}(\mathbb{R}^+), \gamma)$ for probability measure γ corresponding to the Gamma distribution and $k \in (\mathbb{N}, 2^\mathbb{N}, \mu)$ for counting measure μ. To assumption (1), probability measures and the counting measure on $\mathbb{N}$ are both known to be $\sigma-$finite by definition. To assumption (2), we have already demonstrated

that $f(h_j, k)$ in both cases is non-negative using the properties of zonal polynomials for positive semi-definite matrices. For the final assumption (3), note that it suffices to show that the $f(h_j, k)$ are Borel-measurable for fixed k (see Bartle (1995) chapter 10). This follows directly from the fact that each term will reduce to an exponential polynomial in h_j, which are continuous and thus measurable. With this in mind, we proceed:

$$\int_0^\infty \cdots \int_0^\infty \int_{\mathbf{\Psi} \in \mathcal{V}_{K,Q}} g(\mathbf{\Psi}, \mathbf{H}) = \frac{1}{V} \sum_{k=0}^{\infty} \frac{1}{(2k)!} \sum_{|\kappa|=2k} \frac{C_\kappa(\mathbf{P}_\alpha/2)}{C_\kappa(I_K)} \times \int_0^\infty \cdots \int_0^\infty C_\kappa(\mathbf{H}) \prod_{i=1}^{K} h_i^{Q/2} \Gamma(h_i|\alpha_\psi, \beta_\psi)$$
$$- \frac{1}{V} \sum_{k=0}^{\infty} \frac{1}{(2k+1)!} \sum_{|\kappa|=2k+1} \frac{C_\kappa(\mathbf{P}_\alpha/2)}{C_\kappa(I_K)} \times \int_0^\infty \cdots \int_0^\infty C_\kappa(\mathbf{H}) \prod_{i=1}^{K} h_i^{Q/2} \Gamma(h_i|\alpha_\psi, \beta_\psi)$$

At this point, we are able to combine the difference back into a single sum, resulting in the following:

$$\int_0^\infty \cdots \int_0^\infty \int_{\mathbf{\Psi} \in \mathcal{V}_{K,Q}} g(\mathbf{\Psi}, \mathbf{H}) = \frac{1}{V} \sum_{k=0}^{\infty} \frac{(-1)^k}{k!} \sum_{|\kappa|=k} \frac{C_\kappa(\mathbf{P}_\alpha/2)}{C_\kappa(I_K)} \times \int_0^\infty \cdots \int_0^\infty C_\kappa(\mathbf{H}) \prod_{i=1}^{K} h_i^{Q/2} \Gamma(h_i|\alpha_\psi, \beta_\psi)$$

To prove that this alternating series converges, it is sufficient to prove absolute convergence. We define the series of the absolute values below using $S_A(\alpha_\psi, \beta_\psi)$ – a function of the smoothing prior parameters α_ψ, β_ψ. If this series converges, then the integral $\int_0^\infty \cdots \int_0^\infty \int_{\mathbf{\Psi} \in \mathcal{V}_{K,Q}} g(\mathbf{\Psi}, \mathbf{H})$ will be finite as desired.

$$S_A(\alpha_\psi, \beta_\psi) = \frac{1}{V} \sum_{k=0}^{\infty} \frac{1}{k!} \sum_{|\kappa|=k} \frac{C_\kappa(\mathbf{P}_\alpha/2)}{C_\kappa(I_K)} \times \int_0^\infty \cdots \int_0^\infty C_\kappa(\mathbf{H}) \prod_{i=1}^{K} h_i^{Q/2} \Gamma(h_i|\alpha_\psi, \beta_\psi)$$

We can now apply inequality 5.26 from Bagyan and Richards (2024), derived from Faraut and Korányi (1994). This step will provide an upper bounding series for the non-negative $S_A(\alpha_\psi, \beta_\psi)$ which is easier to work with. This inequality states that, for Σ being a $d \times d$ positive definite matrix with ordered eigenvalues $\sigma_1 \geq \ldots \geq \sigma_d$, it follows that

$$C_\kappa(\Sigma) \leq C_\kappa(I_d) \prod_{j=1}^{d} \sigma_j^{\kappa_j}$$

where the partition κ is permitted to contain zero entries. The diagonal matrix $\mathbf{H}$ will have eigenvalues equal to its diagonal elements h_k, which will be non-zero with probability 1, meaning

$\mathbf{H}$ will be positive definite with probability 1. This almost-sure inequality of the integrands will produce the proper inequality when taking the requisite expectation/integral.

$$S_A(\alpha_\psi, \beta_\psi) \leq \frac{1}{V} \sum_{k=0}^{\infty} \frac{1}{k!} \sum_{|\kappa|=k} \frac{C_\kappa(I_K) C_\kappa(\mathbf{P}_\alpha/2)}{C_\kappa(I_K)} \times \int_0^\infty \cdots \int_0^\infty \prod_{i=1}^{K} h_i^{\kappa_i + Q/2} \Gamma(h_i | \alpha_\psi, \beta_\psi)$$
$$= \frac{1}{V} \sum_{k=0}^{\infty} \frac{1}{k!} \sum_{|\kappa|=k} C_\kappa(\mathbf{P}_\alpha/2) \prod_{i=1}^{K} \mathbb{E}[h_i^{\kappa_i + Q/2}]$$

We have reduced the integral of interest to the independent product of moments of each smoothing coefficient. Applying the known form of these moments in terms of the shape α_ψ and rate β_ψ results in the following:

$$S_A(\alpha_\psi, \beta_\psi) \leq \frac{1}{V} \sum_{k=0}^{\infty} \frac{1}{k!} \sum_{|\kappa|=k} C_\kappa(\mathbf{P}_\alpha/2) \prod_{i=1}^{K} \frac{\Gamma(\alpha_\psi + \kappa_i + Q/2)}{\beta_\psi^{\kappa_i + Q/2} \Gamma(\alpha_\psi)}$$
$$= \frac{1}{V} \sum_{k=0}^{\infty} \frac{1}{k! \times \beta_\psi^{k + KQ/2}} \sum_{|\kappa|=k} C_\kappa(\mathbf{P}_\alpha/2) \prod_{i=1}^{K} \prod_{j=1}^{\kappa_i + Q/2} (\alpha_\psi + j - 1)$$

We will now consider the last factor in the above series, for which we provide the shorthand $Pr(\kappa) = \prod_{i=1}^{K} \prod_{j=1}^{\kappa_i + Q/2} (\alpha_\psi + j - 1)$. We endeavor to demonstrate that $Pr(\kappa)$ is maximized by the partition which places all of the weight on a single entry, denoted $\kappa = (k)$ in literature. Towards this end, we will demonstrate that $Pr(\kappa)$ for $\kappa \neq (k)$ is always strictly increased by moving weight to the first entry from the last non-zero entry. Before proceeding, it is important to recall that each κ is defined such that the entries are non-decreasing: $\kappa_1 \geq \kappa_2 \geq \ldots \geq \kappa_K \geq 0$.

Consider an arbitrary integer partition $\kappa \neq (k)$ with length (number of non-zero components) $\mathcal{L}(\kappa)$. We compare $Pr(\kappa)$ with $Pr(\kappa')$ for $\kappa'_1 = \kappa_1 + 1, \kappa'_i = \kappa_i$ when $1 < i < \mathcal{L}(\kappa)$, and $\kappa'_{\mathcal{L}(\kappa)} = \kappa_{\mathcal{L}(\kappa)} - 1$. This κ' still has the same weight, and should satisfy the ordering requirement, so it is a well-defined partition. We can now calculate $\frac{Pr(\kappa')}{Pr(\kappa)}$ as follows. This ratio will always

be well-defined, as $Pr(\cdot)$ will always be a product of positive factors.

$$\begin{aligned}\frac{Pr(\kappa')}{Pr(\kappa)} &= \frac{\prod_{i=1}^{K}\prod_{j=1}^{\kappa'_i+Q/2}(\alpha_\psi+j-1)}{\prod_{i=1}^{K}\prod_{j=1}^{\kappa_i+Q/2}(\alpha_\psi+j-1)} \\ &= \frac{\prod_{j=1}^{(\kappa_1+1)+Q/2}(\alpha_\psi+j-1)}{\prod_{j=1}^{\kappa_1+Q/2}(\alpha_\psi+j-1)} \times \frac{\prod_{j=1}^{(\kappa_{\mathcal{L}(\kappa)}-1)+Q/2}(\alpha_\psi+j-1)}{\prod_{j=1}^{\kappa_{\mathcal{L}(\kappa)}+Q/2}(\alpha_\psi+j-1)} \\ &= \frac{\alpha_\psi+\kappa_1+Q/2}{\alpha_\psi+\kappa_{\mathcal{L}(\kappa)}+Q/2-1}\end{aligned}$$

As $\kappa_1 \geq \kappa_{\mathcal{L}(\kappa)}$ inherently, it follows that the above ratio will be > 1, so $Pr(\cdot)$ is strictly increased by moving weight to the first entry. Then, beginning at any arbitrary partition, transitioning weight from the last non-zero entry in the partition to the first will always increase $Pr(\cdot)$. As any partition for can be related to (k) through a series of such "transitions", it follows from the transitive property that $Pr[(k)] > Pr(\kappa)$ for arbitrary partition κ.

Using the above result, we establish another bounding Series on $S_A(\alpha_\psi, \beta_\psi)$. Through this step, we are able to move the $Pr(\cdot)$ term out of the summation over partitions of weight k, allowing use of the identity $\sum_{|\kappa|=k} C_\kappa(A) = \text{tr}(A)^k$ (Bagyan and Richards, 2024).

$$\begin{aligned}S_A(\alpha_\psi,\beta_\psi) &\leq \frac{1}{V}\sum_{k=0}^{\infty}\frac{1}{k!\times\beta_\psi^{k+KQ/2}}\sum_{|\kappa|=k}C_\kappa(\mathbf{P}_\alpha/2)Pr(\kappa) \\ &< \frac{1}{V}\sum_{k=0}^{\infty}\frac{1}{k!\times\beta_\psi^{k+KQ/2}}\sum_{|\kappa|=k}C_\kappa(\mathbf{P}_\alpha/2)Pr[(k)] \\ &= \frac{1}{V}\sum_{k=0}^{\infty}\frac{1}{k!\times\beta_\psi^{k+KQ/2}}\left[\sum_{|\kappa|=k}C_\kappa(\mathbf{P}_\alpha/2)\right]Pr[(k)] \\ &= \frac{1}{V}\sum_{k=0}^{\infty}\frac{1}{k!\times\beta_\psi^{k+KQ/2}}\text{tr}(\mathbf{P}_\alpha/2)^k Pr[(k)] \\ &= \frac{1}{V}\sum_{k=0}^{\infty}\frac{1}{k!\times\beta_\psi^{k+KQ/2}\times 2^k}\text{tr}(\mathbf{P}_\alpha)^k Pr[(k)]\end{aligned}$$

We can now discern under what conditions this bounding series, and thus the original series

by comparison test, converge. This is accomplished below using the ratio test.

$$\begin{aligned}
a_k &= \frac{1}{k! \times \beta_\psi^{k+KQ/2} \times 2^k} \text{tr}(\mathbf{P}_\alpha)^k Pr[(k)] \\
\implies \frac{a_{k+1}}{a_k} &= \frac{\frac{1}{(k+1)! \times \beta_\psi^{(k+1)+KQ/2} \times 2^{k+1}} \text{tr}(\mathbf{P}_\alpha)^{(k+1)} Pr[(k+1)]}{\frac{1}{k! \times \beta_\psi^{k+KQ/2} \times 2^k} \text{tr}(\mathbf{P}_\alpha)^k Pr[(k)]} \\
&= \frac{\text{tr}(\mathbf{P}_\alpha)}{2(k+1)\beta_\psi} \times \frac{\prod_{j=1}^{(k+1)+Q/2}(\alpha_\psi + j - 1) \times \prod_{i=2}^{K} \prod_{p=1}^{Q/2}(\alpha_\psi + p - 1)}{\prod_{j'=1}^{k+Q/2}(\alpha_\psi + j' - 1) \times \prod_{i'=2}^{K} \prod_{p'=1}^{Q/2}(\alpha_\psi + p' - 1)} \\
&= \frac{\text{tr}(\mathbf{P}_\alpha)(\alpha_\psi + k + Q/2)}{2(k+1)\beta_\psi}
\end{aligned}$$

Taking the limit as $k \to \infty$ provides the following.

$$\begin{aligned}
\lim_{k\to\infty} \frac{a_{k+1}}{a_k} &= \lim_{k\to\infty} \frac{\text{tr}(\mathbf{P}_\alpha)(\alpha_\psi + k + Q/2)}{2(k+1)\beta_\psi} \\
&= \frac{\text{tr}(\mathbf{P}_\alpha)}{2\beta_\psi} \lim_{k\to\infty} \frac{\alpha_\psi + k + Q/2}{k+1} \\
&= \frac{\text{tr}(\mathbf{P}_\alpha)}{2\beta_\psi}
\end{aligned}$$

By the ratio test, the above series a_k converges if and only if $\lim_{k\to\infty} \frac{a_{k+1}}{a_k} < 1$. This occurs precisely when $\frac{\text{tr}(\mathbf{P}_\alpha)}{2\beta_\psi} < 1 \implies \text{tr}(\mathbf{P}_\alpha)/2 < \beta_\psi$. This informs our choice of the prior parameter β_ψ, as it must be greater than the half of the trace (or half of the sum of the eigenvalues) for the penalty matrix. This makes some intuitive sense, as the smoothing parameters having overly heavy upper tails, combined with there being non-trivial regions of the manifold $\mathcal{V}_{K,Q}$ over which penalization is quite extreme (quantified through the eigenvalues themselves), could result in an unbounded integrated penalty.

3 Joint and conditional posteriors

We begin by enumerating the model likelihood and parameter priors explicitly. We use $\Gamma(a, b)$ to denote the gamma distribution with shape a and rate b, $\Gamma^{-1}(a, b)$ to denote the inverse gamma distribution with shape a and scale b, $N(\mu, \sigma^2)$ to indicate the normal distribution with mean μ and variance σ^2, and $MVN(\boldsymbol{\mu}, \boldsymbol{\Sigma})$ to denote the multivariate normal distribution with mean $\boldsymbol{\mu}$ and variance-covariance $\boldsymbol{\Sigma}$. Throughout, I_A denotes the identity matrix of dimension A.

Variance Component Priors:

Prior distributions of the variance and smoothing parameter components are as follows.

$$\sigma^2 \sim \Gamma^{-1}(\alpha_\sigma, \beta_\sigma)$$
$$\lambda_k \sim \Gamma^{-1}(\alpha_\lambda, \beta_\lambda) \quad \forall k = 1, \ldots, K$$
$$h_\mu \sim \Gamma(\alpha_\mu, \beta_\mu)$$
$$h_k \sim \Gamma(\alpha_\psi, \beta_\psi) \quad \forall k = 1, \ldots, K$$

We set nearly uninformative priors, $\alpha_\sigma, \alpha_\lambda, \alpha_\psi, \alpha_\mu = 0.001$ and $\beta_\sigma, \beta_\lambda, \beta_\psi, \beta_\mu = 0.001$.

We also enforce ordering of the eigenvalues, requiring the indicator $\mathbb{1}(\lambda_1 \geq \ldots \geq \lambda_K)$.

Prior on w_μ:

FAST implements a smoothing prior on $\mu(t)$ through a posterior penalty on spline coefficients w_μ. For prior density $f(w_\mu)$, we have the following, where $\mathbf{R}(\cdot)$ indicates matrix rank.

$$f(w_\mu) \propto h_\mu^{\mathbf{R}(\mathbf{P}_\alpha)/2} \exp\left\{-\frac{h_\mu}{2} w_\mu^t \mathbf{P}_\alpha w_\mu\right\}$$

Prior on FPCs:

FAST implements a similar smoothing prior on the $\phi_k(t)$ through the spline coefficients ψ_k, For prior density $f(\psi_k)$, we have the following, where $\mathbf{R}(\cdot)$ indicates matrix rank.

$$f(\psi_k) \propto h_k^{\mathrm{R}(\mathbf{P}_\alpha)/2} \exp\left\{-\frac{h_k}{2} \psi_k^t \mathbf{P}_\alpha \psi_k\right\}$$

For orthonormality of the FPCs, FAST requires the additional constraint that the matrix $\boldsymbol{\Psi} = [\psi_1|\ldots|\psi_K]$ is orthonormal. This requires introducing the indicator $\mathbb{1}(\boldsymbol{\Psi} \in \mathcal{V}_{K,Q})$ to the prior distribution, where $\mathcal{V}_{K,Q}$ is the Stiefel manifold of dimension $Q \times K$.

Prior on the Scores:

Score priors are as defined in FPCA according to the Kosambi-Karhunen-Loève decomposition: $\xi_{ik} \sim N(0, \lambda_k)$ for $i \in \{1, 2, \ldots, N\}$ and $k \in \{1, 2, \ldots, K\}$.

Model Likelihood:

We begin with notation. Let $Y_i \in \mathbb{R}^M$ represent the vector of data for participant i observed at the times $t \in \{t_1, \ldots, t_M\}$. Correspondingly, let $\mathbf{B} \in \mathbb{R}^{M \times Q}$ be the orthonormal basis matrix evaluated at the same set of observation points. Using these definitions, the FPCA model likelihood contribution is as follows.

$$Y_i \sim MVN\{\mathbf{B}(w_\mu + \sum_{k=1}^{K} \xi_{ik}\psi_k), \sigma^2 I_M\}$$

Joint Posterior:

Given the likelihood and priors previously described, the posterior density of all model parameters given the data is proportional to the following. Throughout, we use the notation $f(X|\Theta)$ to represent the evaluation of density $f(\cdot)$ at point X with parameters Θ.

$$\begin{aligned}
&\prod_{i=1}^{N} MVN\{Y_i|\mathbf{B}(w_\mu + \sum_{k=1}^{K} \xi_{ik}\psi_k), \sigma^2 I_M\} \times \Gamma^{-1}(\sigma^2|\alpha_\sigma, \beta_\sigma) \\
&\times \left\{ \prod_{k=1}^{K} N(\xi_{ik}|0, \lambda_k) \times \Gamma^{-1}(\lambda_k|\alpha_\lambda, \beta_\lambda) \times h_k^{\mathbf{R}(\mathbf{P}_\alpha)/2} \exp\left(-\frac{h_k}{2}\psi_k^t \mathbf{P}_\alpha \psi_k\right) \times \Gamma(h_k|\alpha_\psi, \beta_\psi) \right\} \quad (1) \\
&\times h_\mu^{\mathbf{R}(\mathbf{P}_\alpha)/2} \exp\left(-\frac{h_\mu}{2} w_\mu^t \mathbf{P}_\alpha w_\mu\right) \times \Gamma(h_\mu|\alpha_\mu, \beta_\mu) \times \mathbb{1}(\lambda_1 \geq \ldots \geq \lambda_K) \times \mathbb{1}(\mathbf{\Psi} \in \mathcal{V}_{Q,K})
\end{aligned}$$

Using this joint posterior form, we begin to derive the individual conditional posteriors for each component.

Smoothing Parameters:

First, we derive the conditional posterior for the smoothing parameter for the mean, h_μ, which we denote $f(h_\mu|\text{others})$.

$$\begin{aligned}
f(h_\mu|\text{others}) &\propto h_\mu^{\mathbf{R}(\mathbf{P}_\alpha)/2} \exp\left(-\frac{h_\mu}{2} w_\mu^t \mathbf{P}_\alpha w_\mu\right) \times \Gamma(h_\mu|\alpha_\mu, \beta_\mu) \\
&\propto h_\mu^{\mathbf{R}(\mathbf{P}_\alpha)/2} \exp\left(-\frac{h_\mu}{2} w_\mu^t \mathbf{P}_\alpha w_\mu\right) \times h_\mu^{\alpha_\mu - 1} \exp(-\beta_\mu h_\mu) \\
&\propto h_\mu^{\{\mathbf{R}(\mathbf{P}_\alpha)/2 + \alpha_\mu\} - 1} \exp\{-\left(\frac{w_\mu^t \mathbf{P}_\alpha w_\mu}{2} + \beta_\mu\right) h_\mu\}
\end{aligned}$$

One can recognize the form of the gamma distribution in the last line above.

$$[h_\mu|\text{others}] \sim \Gamma(\mathbf{R}(\mathbf{P}_\alpha)/2 + \alpha_\mu, w_\mu^t \mathbf{P}_\alpha w_\mu/2 + \beta_\mu)$$

We can similarly derive the conditional posterior distribution for the general eigenfunction smoothing parameter h_k, which we denote $f(h_k|\text{others})$.

$$\begin{aligned} f(h_k|\text{others}) &\propto h_k^{\mathbf{R}(\mathbf{P}_\alpha)/2} \exp\left(-\frac{h_k}{2}\psi_k^t \mathbf{P}_\alpha \psi_k\right) \times \Gamma(h_k|\alpha_\psi, \beta_\psi) \\ &\propto h_k^{\mathbf{R}(\mathbf{P}_\alpha)/2} \exp\left(-\frac{h_k}{2}\psi_k^t \mathbf{P}_\alpha \psi_k\right) \times h_k^{\alpha_\psi - 1} \exp(-\beta_\psi h_k) \\ &\propto h_k^{\{\mathbf{R}(\mathbf{P}_\alpha)/2 + \alpha_\psi\} - 1} \exp\{-\left(\frac{\psi_k^t \mathbf{P}_\alpha \psi_k}{2} + \beta_\psi\right) h_k\} \end{aligned}$$

Once more, the form of the gamma distribution is clear in the final line above.

$$[h_k|\text{others}] \sim \Gamma(\mathbf{R}(\mathbf{P}_\alpha)/2 + \alpha_\psi, \psi_k^t \mathbf{P}_\alpha \psi_k/2 + \beta_\psi)$$

<u>Noise variance σ^2:</u>

We derive the conditional posterior of σ^2, denoted $f(\sigma^2|\text{others})$, below.

$$\begin{aligned} f(\sigma^2|\text{others}) &\propto \prod_{i=1}^N MVN\{Y_i|\mathbf{B}(w_\mu + \sum_{k=1}^K \xi_{ik}\psi_k), \sigma^2 I_M\} \times \Gamma^{-1}(\sigma^2|\alpha_\sigma, \beta_\sigma) \\ &\propto \prod_{i=1}^N (\sigma^2)^{-M/2} \exp(-\frac{||Y_i - \mathbf{B}(w_\mu + \sum_{k=1}^K \xi_{ik}\psi_k)||^2}{2\sigma^2}) \times (\sigma^2)^{-\alpha_\sigma - 1} \exp(-\frac{\beta_\sigma}{\sigma^2}) \\ &\propto (\sigma^2)^{-(NM/2 + \alpha_\sigma) - 1} \exp\{-\frac{1}{\sigma^2}\left(\frac{1}{2}\sum_{i=1}^n ||Y_i - \mathbf{B}(w_\mu + \sum_{k=1}^K \xi_{ik}\psi_k)||^2 + \beta_\sigma\right)\} \end{aligned}$$

The last line above is clearly the form of the inverse gamma distribution, with exact parameterization as follows:

$$[\sigma^2|\text{others}] \sim \Gamma^{-1}(\frac{NM}{2} + \alpha_\sigma, \frac{1}{2}\sum_{i=1}^n ||Y_i - \mathbf{B}(w_\mu + \sum_{k=1}^K \xi_{ik}\psi_k)||^2 + \beta_\sigma])$$

<u>Eigenvalues λ_k:</u>

The joint conditional posterior distribution of the eigenvalaues λ_k, denoted $f(\lambda_1, \ldots, \lambda_K|\text{others})$,

is derived below.

$$
\begin{aligned}
f(\lambda_1, \ldots, \lambda_K|\text{others}) &\propto \prod_{k=1}^{K}\prod_{i=1}^{N} N(\xi_{ik}|0, \lambda_k) \times \Gamma^{-1}(\lambda_k|\alpha_\lambda, \beta_\lambda) \times \mathbb{1}(\lambda_1 \geq \ldots \geq \lambda_K) \\
&\propto \prod_{k=1}^{K} \lambda_k^{-N/2} \exp(-\frac{1}{2\lambda_k}\sum_{i=1}^{N}\xi_{ik}^2) \times \lambda_k^{-\alpha_\lambda - 1}\exp(-\frac{\beta_\lambda}{\lambda_k}) \times \mathbb{1}(\lambda_1 \geq \ldots \geq \lambda_K) \\
&\propto \prod_{k=1}^{K} \lambda_k^{-(N/2+\alpha_\lambda)-1} \exp\{-\frac{1}{\lambda_k}\left(\frac{1}{2}\sum_{i=1}^{N}\xi_{ik}^2 + \beta_\lambda\right)\} \times \mathbb{1}(\lambda_1 \geq \ldots \geq \lambda_K)
\end{aligned}
$$

The above joint distribution has the form of independent inverse gamma distributions ($[\lambda_k|\text{others}] \sim \Gamma^{-1}(N/2 + \alpha_\lambda, \frac{1}{2}\sum_{i=1}^{N}\xi_{ik}^2 + \beta_\lambda)$), with the additional constraint that the λ_k be ordered. Sampling from this type of joint distribution is possible using ordered transforms and corresponding Jacobian transforms as described in the STAN documentation (Team, 2025).

Mean spline coefficients w_μ:

We derive the conditional posterior of w_μ, denoted $f(w_\mu|\text{others})$ as follows.

$$
\begin{aligned}
f(w_\mu|\text{others}) &\propto \prod_{i=1}^{N} MVN\{Y_i|\mathbf{B}(w_\mu + \sum_{k=1}^{K}\xi_{ik}\psi_k), \sigma^2 I_M\} \times \exp\left(-\frac{h_\mu}{2}w_\mu^t\mathbf{P}_\alpha w_\mu\right) \\
&\propto \prod_{i=1}^{N} \exp\left\{-\frac{||\mathbf{B}w_\mu - (Y_i - \mathbf{B}\sum_{k=1}^{K}\xi_{ik}\psi_k)||^2}{2\sigma^2} - \frac{h_\mu}{2}w_\mu^t\mathbf{P}_\alpha w_\mu\right\} \\
&\propto \exp\left\{-\sum_{i=1}^{N}\frac{||\mathbf{B}w_\mu||^2 - 2\langle Y_i - \mathbf{B}\sum_{k=1}^{K}\xi_{ik}\psi_k, \mathbf{B}w_\mu\rangle}{2\sigma^2} - \frac{h_\mu}{2}w_\mu^t\mathbf{P}_\alpha w_\mu\right\}
\end{aligned}
$$

We now introduce the shorthand $D_i = Y_i - \mathbf{B}\sum_{k=1}^{K}\xi_{ik}\psi_k$ to denote the residual between the data Y_i and the cumulative random effect $\mathbf{B}\sum_{k=1}^{K}\xi_{ik}\psi_k$. We continue using this notation.

$$
\begin{aligned}
f(w_\mu|\text{others}) &\propto \exp\left\{-\sum_{i=1}^{N}\frac{||\mathbf{B}w_\mu||^2 - 2D_i^t\mathbf{B}w_\mu}{2\sigma^2} - w_\mu^t\frac{h_\mu\mathbf{P}_\alpha}{2}w_\mu\right\} \\
&\propto \exp\left\{-w_\mu^t\left(\sum_{i=1}^{N}\frac{\mathbf{B}^t\mathbf{B}}{2\sigma^2}\right)w_\mu + \left(\sum_{i=1}^{N}\frac{D_i^t\mathbf{B}}{\sigma^2}\right)w_\mu - w_\mu^t\frac{h_\mu\mathbf{P}_\alpha}{2}w_\mu\right\} \\
&\propto \exp\left\{-w_\mu^t\left(\frac{N\mathbf{B}^t\mathbf{B}}{2\sigma^2} + \frac{h_\mu\mathbf{P}_\alpha}{2}\right)w_\mu + \left(\sum_{i=1}^{N}\frac{D_i^t\mathbf{B}}{\sigma^2}\right)w_\mu\right\} \\
&\propto \exp\left[-\frac{1}{2}\{w_\mu^t\left(\frac{N\mathbf{B}^t\mathbf{B}}{\sigma^2} + h_\mu\mathbf{P}_\alpha\right)w_\mu - 2\left(\sum_{i=1}^{N}\frac{D_i^t\mathbf{B}}{\sigma^2}\right)w_\mu\}\right]
\end{aligned}
$$

Completing the square, we find that w_μ has a multivariate normal distribution

$$[w_\mu|\text{others}] \sim MVN\{\left(\frac{N\mathbf{B}^t\mathbf{B}}{\sigma^2} + h_\mu\mathbf{P}_\alpha\right)^{-1}\frac{1}{\sigma^2}\sum_{i=1}^{N}\mathbf{B}^tD_i, \left(\frac{N\mathbf{B}^t\mathbf{B}}{\sigma^2} + h_\mu\mathbf{P}_\alpha\right)^{-1}\}$$

Scores ξ_{ik}:

For arbitrary score ξ_{ik} (study participant i and FPC k), we derive the conditional posterior $f(\xi_{ik}|\text{others})$ as follows.

$$\begin{aligned} f(\xi_{ik}|\text{others}) &\propto MVN\{Y_i|\mathbf{B}(w_\mu + \sum_{p=1}^{K}\xi_{ip}\psi_p), \sigma^2 I_M\} \times N(\xi_{ik}|0, \lambda_k) \\ &\propto \exp(-\frac{||Y_i - \mathbf{B}(w_\mu + \sum_{p=1}^{K}\xi_{ip}\psi_p)||^2}{2\sigma^2} - \frac{\xi_{ik}^2}{2\lambda_k}) \\ &\propto \exp(-\frac{||Y_i - \mathbf{B}(w_\mu + \sum_{p\neq k}^{K}\xi_{ip}\psi_p) - \mathbf{B}\psi_k\xi_{ik}||^2}{2\sigma^2} - \frac{\xi_{ik}^2}{2\lambda_k}) \end{aligned}$$

For the sake of conciseness, we now define the residual quantity $P_{ik} = Y_i - \mathbf{B}(w_\mu + \sum_{p\neq k}^{K}\xi_{ip}\psi_p)$, which can be thought of as the residual between $Y_i - \mathbf{B}w_\mu$ and the projection of $Y_i - \mathbf{B}w_\mu$ onto the FPCs other than k.

$$\begin{aligned} f(\xi_{ik}|\text{others}) &\propto \exp(-\frac{||P_{ik} - \mathbf{B}\psi_k\xi_{ik}||^2}{2\sigma^2} - \frac{\xi_{ik}^2}{2\lambda_k}) \\ &\propto \exp(-\frac{||\mathbf{B}\psi_k||^2\xi_{ik}^2 - 2P_{ik}^t\mathbf{B}\psi_k\xi_{ik}}{2\sigma^2} - \frac{\xi_{ik}^2}{2\lambda_k}) \\ &\propto \exp[-\frac{1}{2}\left\{\xi_{ik}^2\left(\frac{||\mathbf{B}\psi_k||^2}{\sigma^2} + \frac{1}{\lambda_k}\right) - 2\frac{P_{ik}^t\mathbf{B}\psi_k}{\sigma^2}\xi_{ik}\right\}] \end{aligned}$$

Completing the square, we find that ξ_{ik} has a normal distribution.

$$[\xi_{ik}|\text{others}] \sim N\{\left(\frac{||\mathbf{B}\psi_k||^2}{\sigma^2} + \frac{1}{\lambda_k}\right)^{-1}\frac{P_{ik}^t\mathbf{B}\psi_k}{\sigma^2}, \left(\frac{||\mathbf{B}\psi_k||^2}{\sigma^2} + \frac{1}{\lambda_k}\right)^{-1}\}$$

FPC Weights $\mathbf{\Psi}$:

We derive the posterior distribution of the full matrix of FPC spline weights, denoted

$f(\mathbf{\Psi}|\text{others})$, below.

$$f(\mathbf{\Psi}|\text{others}) \propto \prod_{i=1}^{N} MVN\{Y_i|\mathbf{B}(w_\mu + \sum_{k=1}^{K} \xi_{ik}\psi_k), \sigma^2 I_M\} \times \prod_{k=1}^{K} h_k^{\mathbf{R}(\mathbf{P}_\alpha)/2} \exp\left(-\frac{h_k}{2}\psi_k^t \mathbf{P}_\alpha \psi_k\right) \times \mathbb{1}(\mathbf{\Psi} \in \mathcal{V}_{K,Q})$$
$$\propto \exp(-\frac{1}{2\sigma^2}\sum_{i=1}^{N}||Y_i - \mathbf{B}(w_\mu + \sum_{k=1}^{K}\xi_{ik}\psi_k)||^2) \times \exp(-\frac{1}{2}\sum_{k=1}^{K} h_k \psi_k^t \mathbf{P}_\alpha \psi_k) \times \mathbb{1}(\mathbf{\Psi} \in \mathcal{V}_{K,Q})$$

We now introduce the shorthand notation: score vector $\xi_i = \{\xi_{i1}, \ldots, \xi_{iK}\}^t \in \mathbb{R}^K$, diagonal smoothing parameter matrix $\mathbf{H} = \text{diag}(h_1, \ldots, h_K)$, and residual vector $R_i = Y_i - \mathbf{B}w_\mu$. Let $\text{tr}(\cdot)$ indicate the trace of a matrix.

$$f(\mathbf{\Psi}|\text{others}) \propto \exp\left\{-\frac{1}{2\sigma^2}\sum_{i=1}^{N}||R_i - \mathbf{B\Psi}\xi_i||^2 - \frac{1}{2}\text{tr}(\mathbf{H\Psi}^t\mathbf{P}_\alpha\mathbf{\Psi})\right\} \times \mathbb{1}(\mathbf{\Psi} \in \mathcal{V}_{K,Q})$$
$$\propto \exp\left\{-\frac{1}{2\sigma^2}\sum_{i=1}^{N}\left(||R_i||^2 - 2R_i^t\mathbf{B\Psi}\xi_i + ||\mathbf{B\Psi}\xi_i||^2\right) - \frac{1}{2}\text{tr}(\mathbf{H\Psi}^t\mathbf{P}_\alpha\mathbf{\Psi})\right\} \times \mathbb{1}(\mathbf{\Psi} \in \mathcal{V}_{K,Q})$$
$$\propto \exp\left\{-\frac{1}{2\sigma^2}\sum_{i=1}^{N}\left(-2R_i^t\mathbf{B\Psi}\xi_i + ||\mathbf{B\Psi}\xi_i||^2\right) - \frac{1}{2}\text{tr}(\mathbf{H\Psi}^t\mathbf{P}_\alpha\mathbf{\Psi})\right\} \times \mathbb{1}(\mathbf{\Psi} \in \mathcal{V}_{K,Q})$$
$$\propto \exp\left\{-\frac{1}{2\sigma^2}\left(-2\sum_{i=1}^{N}R_i^t\mathbf{B\Psi}\xi_i + \sum_{i=1}^{N}||\mathbf{B\Psi}\xi_i||^2\right) - \frac{1}{2}\text{tr}(\mathbf{H\Psi}^t\mathbf{P}_\alpha\mathbf{\Psi})\right\} \times \mathbb{1}(\mathbf{\Psi} \in \mathcal{V}_{K,Q})$$

We now introduce more notation: score matrix $\Xi \in \mathbb{R}^{K\times N}$, where each row is the score vector ξ_i, and residual matrix $\mathbf{R} \in \mathbb{R}^{N\times M}$, where each row is the residual vector R_i^t.

$$f(\mathbf{\Psi}|\text{others}) \propto \exp\left[-\frac{1}{2\sigma^2}\left\{-2\,\text{tr}(\mathbf{RB\Psi}\Xi) + \text{tr}(\Xi^t\mathbf{\Psi}^t\mathbf{B}^t\mathbf{B\Psi}\Xi)\right\} - \frac{1}{2}\text{tr}\{\mathbf{H\Psi}^t\mathbf{P}_\alpha\mathbf{\Psi}\}\right] \times \mathbb{1}(\mathbf{\Psi} \in \mathcal{V}_{K,Q})$$
$$\propto \exp\left\{\frac{1}{\sigma^2}\text{tr}(\mathbf{RB\Psi}\Xi) - \frac{1}{2\sigma^2}\text{tr}(\Xi^t\mathbf{\Psi}^t\mathbf{B}^t\mathbf{B\Psi}\Xi) - \frac{1}{2}\text{tr}(\mathbf{H\Psi}^t\mathbf{P}_\alpha\mathbf{\Psi})\right\} \times \mathbb{1}(\mathbf{\Psi} \in \mathcal{V}_{K,Q})$$
$$\propto \exp\left\{\frac{1}{\sigma^2}\text{tr}(\Xi\mathbf{RB\Psi}) - \frac{1}{2\sigma^2}\text{tr}(\Xi\Xi^t\mathbf{\Psi}^t\mathbf{B}^t\mathbf{B\Psi}) - \frac{1}{2}\text{tr}(\mathbf{H\Psi}^t\mathbf{P}_\alpha\mathbf{\Psi})\right\} \times \mathbb{1}(\mathbf{\Psi} \in \mathcal{V}_{K,Q})$$
$$\propto \exp\left\{\text{tr}\left(\frac{\Xi\mathbf{RB\Psi}}{\sigma^2} - \frac{\Xi\Xi^t\mathbf{\Psi}^t\mathbf{B}^t\mathbf{B\Psi}}{2\sigma^2} - \frac{\mathbf{H\Psi}^t\mathbf{P}_\alpha\mathbf{\Psi}}{2}\right)\right\} \times \mathbb{1}(\mathbf{\Psi} \in \mathcal{V}_{K,Q})$$

The final line above is the form found in Result 2, which is not of a known distributional family.

4 Simulation results

4.1 Background

All code used for data simulation and subsequent model fitting can be found at this GitHub repository. We compare the data generating model used in S1, meant to imitate the CGM data from DASH4D, to the actual FPCs estimated from the real CGM data in Figure 1.

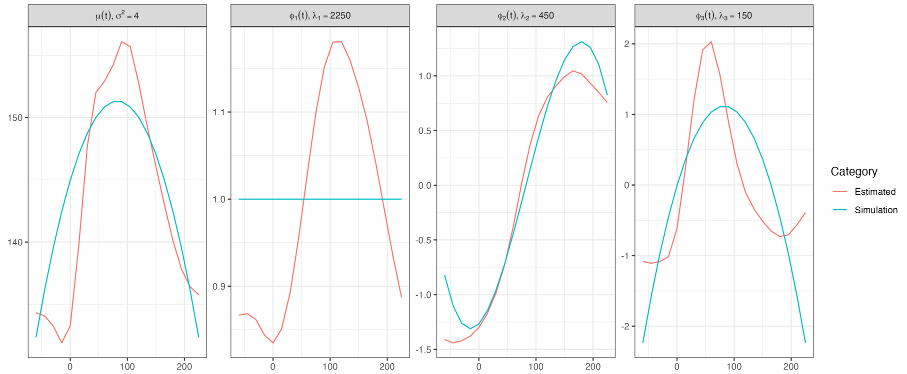

Figure 1: Comparison between the generative FPCs used in simulations ("Simulation") and estimates from fitting FPCA to the CGM data ("Estimated"). The CGM data used was the by-participant mean postprandial response curves from the DASH4D study.

While the FPCs used in our generative model for S1 ("Simulation") differ from the FPCs estimated from the mean postprandial CGM responses ("Estimated") in several non-trivial aspects, we choose them for their balance between adequate approximation and simplicity of generation. We built the "Simulation" bases using linear combinations of the Legendre polynomials, a known orthonormal basis of $L^2([0,1])$, to enhance reproducibility.

4.2 Fixed effects comparison

We compare the fixed effects estimates of $\mu(t)$ produced by FAST to those from GFSR and VMP. POLAR is not included, as it subtracts off the column means from teh data matrix in pre-processing rather than estimating the mean as part of the model. We compare ISE of the posterior estimate and aggregate coverage of equal-tailed 95% credible intervals, each calculated using the same procedures outlined for the FPCs in main text Section 4.

Figure 2 demonstrates the ISE of $\mu(t)$ by method, where row indicates simulation scenario. FAST produces estimates of $\mu(t)$ with ISE similar or superior to those of the comparator methods.

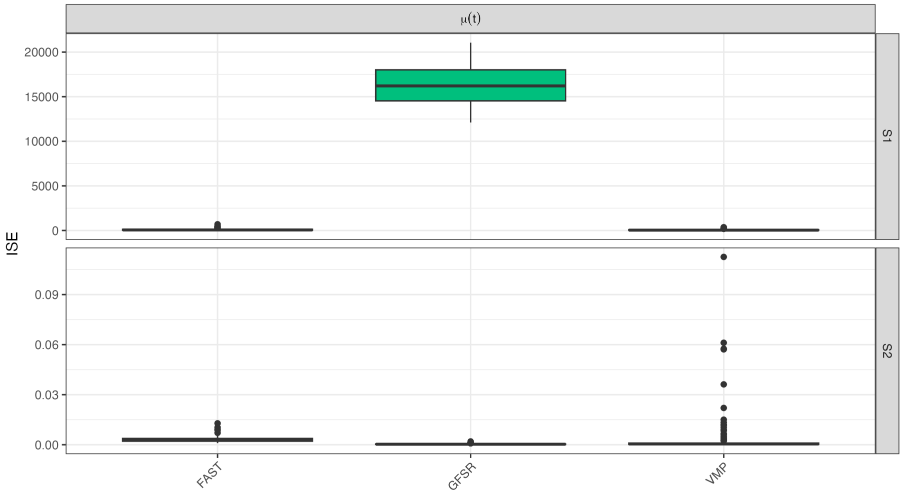

Figure 2: Boxplots of ISE for the mean $\mu(t)$ by estimation method. Row indicates simulation scenario, S1 then S2.

GFSR notably has substantial error for S1. Based upon further analysis of this phenomenon, we believe this is due to projection of the fixed effect onto the two FPCs $\phi_1(t), \phi_3(t)$, as described in main text Section 4.

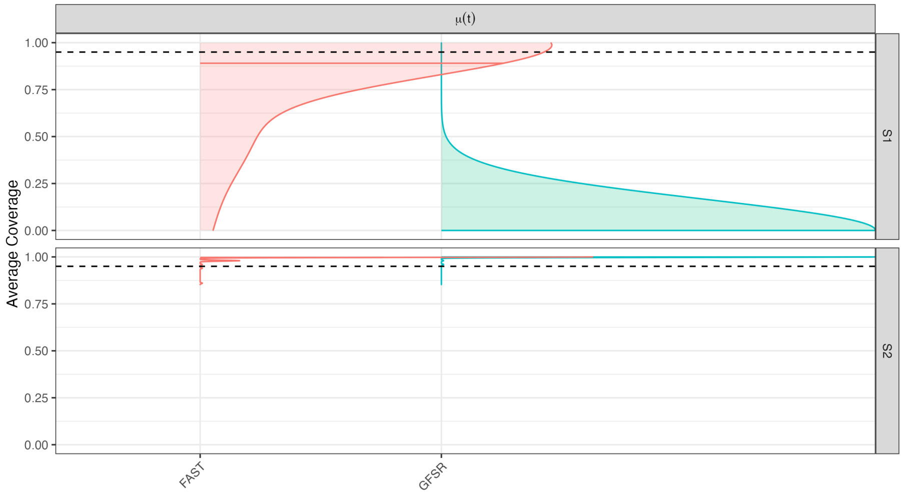

Figure 3: Kernel smoother of coverage probabilities of 95% credible intervals of the true mean $\mu(t)$ for FAST and GFSR. Row indicates simulation scenario, S1 then S2. Distribution means: horizontal solid lines; nominal 95% level: horizontal dotted lines.

Figure 3 visualizes kernel smooths of equal-tailed 95% credible interval coverage for FAST and GFSR, where row again indicates simulation scenario. We do not include POLAR and VMP in this comparison, as neither produces posterior inferences upon the mean function $\mu(t)$. Inference is similar between FAST and GFSR for the canonical scenario S2, but coverage is far

closer to nominal for FAST in the CGM-based S1.

4.3 Score comparison

We also compare FAST to existing Bayesian FPCA implementations in their estimation of the score ξ_{ik}. Metrics of interest include mean squared error (MSE) of posterior mean estimates and mean coverage of equal-tail 95% credible intervals, each aggregated over curves i for each combination of FPC k and simulation.

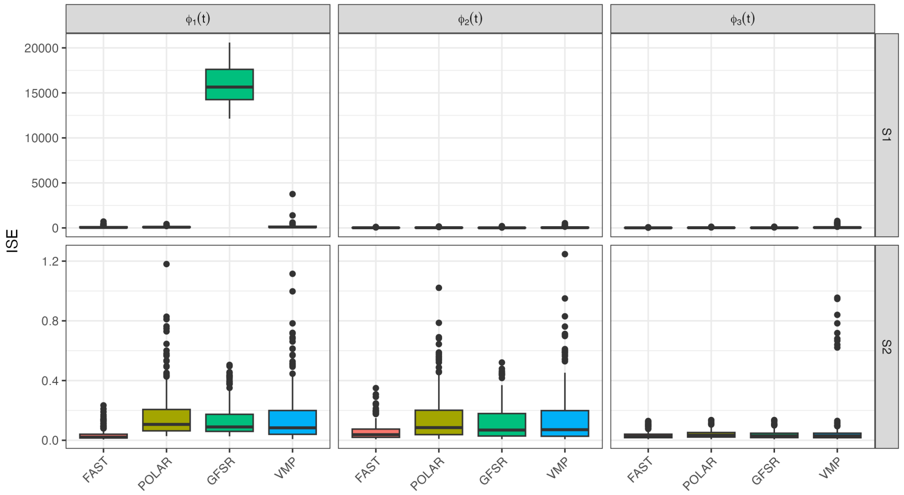

Figure 4: Boxplots of MSE for the scores ξ_{ik} by estimation method. Column indicates FPC, and row indicates simulation scenario, S1 then S2.

Figure 4 visualizes the distribution of score MSE over simulations. FAST is has consistently similar or lower MSE when compared to the existing methods. GFSR has much larger MSE for those scores associated with $\phi_1(t)$ in S1. The order of this MSE is close to $140^2 = 19600$, supporting our previous assertion that the mean $\mu(t)$ is being projected onto $\phi_1(t)$ and $\phi_3(t)$ by this implementation.

Figure 5 displays the kernel smooth of the vectors of coverage proportions for FAST and relevant comparator methods by FPC (columns) and simulation scenario (rows). FAST is the only implementation which produces consistently close to nominal coverage for all FPCs under both scenarios.

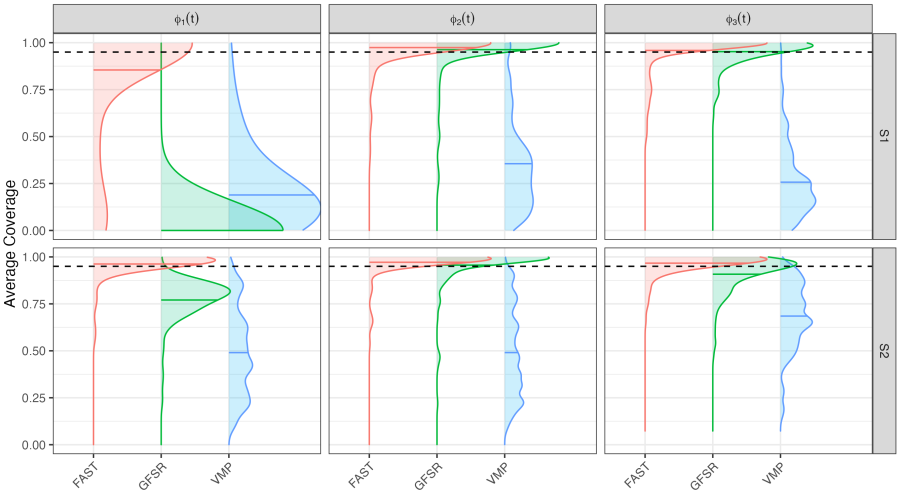

Figure 5: Kernel smoother of coverage probabilities of 95% credible intervals of the true scores, ξ^b_{ik}, for FAST, GFSR, and VMP. First row: S1; second row: S2. Each column: corresponding eigenfunction. Distribution means: horizontal solid lines; nominal 95% level: horizontal dotted lines.

4.4 Multilevel extension

We perform a simple 2-level simulation study to assess performance of the multilevel extension of FAST in comparison to GFSR. This simulation includes 50 groups at the first level ($N = 50$), each with 5 functional observations ($\forall i,\ J_i = 5$) for a total of 250 functions. We are only able to compare against GFSR due to both POLAR and VMP having no available multilevel extension. We use a well-known and frequently used multilevel simulation scenario for our study, where the FPCs are orthogonal within level but not between. All explicit details are provided below (Cui et al., 2023).

Multilevel Scenario

$$\phi^{(1)}_k(t) = \{\sqrt{2}\sin(2\pi t), \sqrt{2}\cos(2\pi t), \sqrt{2}\sin(4\pi t), \sqrt{2}\cos(4\pi t)\}; \quad \lambda^{(1)}_k = 0.5^{k-1}$$

$$\phi^{(2)}_l(t) = \{1, \sqrt{3}(2t-1), \sqrt{5}(6t^2-6t+1), \sqrt{7}(20t^3-30t^2+12t-1)\}; \quad \lambda^{(2)}_l = 0.5^{l-1}$$

$$\mu(t) = 0; \quad \sigma^2 = 1$$

We first present the integrated squared error (ISE) of the FPCs $\phi^{(1)}_k(t), \phi^{(2)}_l(t)$. We calculate this measure using the same procedure detailed in main text Section 4. Each panel corresponds to one of the eigenfunctions, with column indicating index and row indicating level.

Figure 6 indicates uniformly lower ISE for FAST as compared to GFSR. These differences

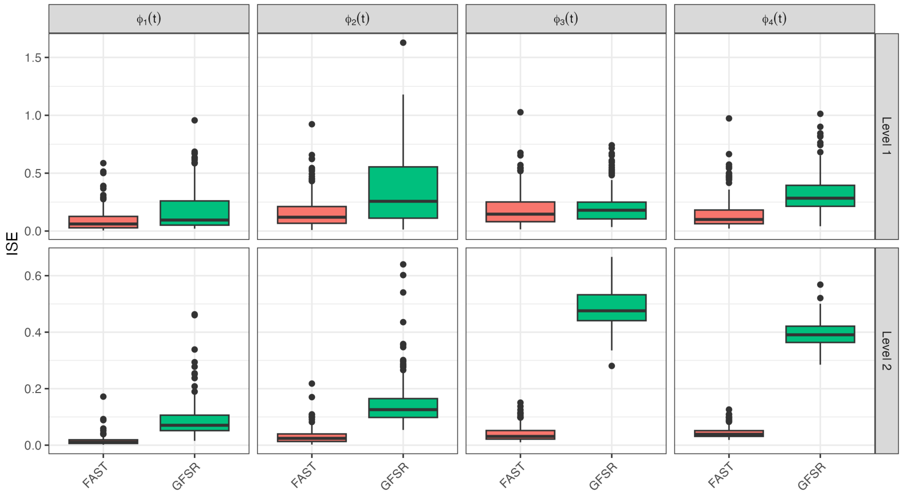

Figure 6: Boxplots of FPC ISE for FAST and GFSR from the multilevel simulations. Row indicates hierarchical level, while columns correspond to FPCs.

can be extreme, see for example the third and fourth FPCs at the visit level, $\phi_3^{(2)}(t), \phi_4^{(2)}(t)$.

We next consider FPC coverage according to the point-wise equal-tailed 95% credible intervals produced by FAST and GFSR. As in main text Section 4, we estimate coverage probability using the proportion of time points which are covered for each simulated dataset, visualizing the result with a kernel smooth in Figure 7. We include on this Figure the mean of each coverage distribution (horizontal solid lines) and the nominal coverage 95% coverage level (horizontal dotted lines).

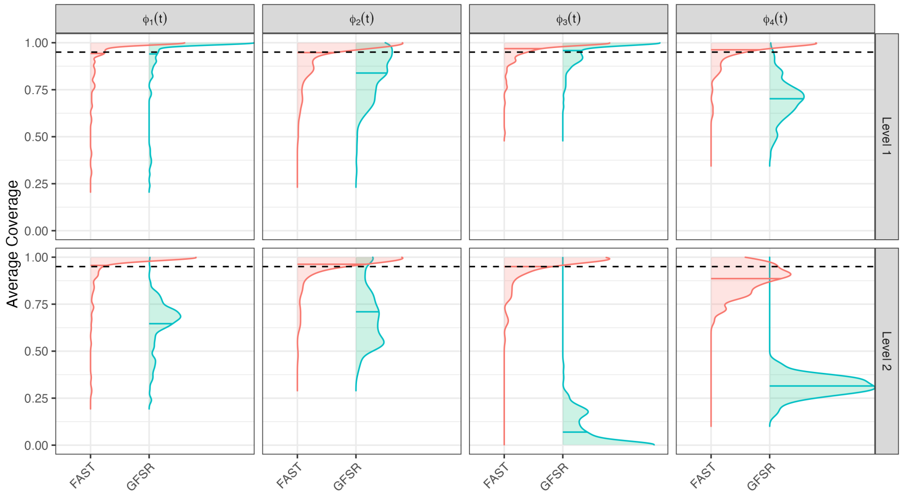

Figure 7: Kernel smoother of coverage probabilities of 95% credible intervals of the true FPCs for FAST and GFSR from the multilevel simulations. Row indicates hierarchical level, while columns correspond to FPCs. The horizontal dotted lines indicate the nominal 95% level.

Similar to the single-level simulations, we find that FAST produces nearly nominal coverage for all FPCs. In comparison, GFSR does not cover the eigenfunctions at the visit level well. For these FPCs, mean coverage ranges from ≈ 0.15 to ≈ 0.7.

This disparity in coverage extends to the scores at both levels, ξ_{ik} and ζ_{ijk}, as can be observed in Figure 8. We estimate score coverage in the same fashion illustrated in main text Section 4. For each simulated dataset $b \leq B$, we calculate the 95% credible intervals for each individual $\xi_{ik}^b, \zeta_{ijk}^b$ from the posterior samples. We then estimate the coverage probability by aggregating the coverage indicators by the corresponding eigenfunction. Figure 8 visualizes the kernel smooths of the corresponding vectors of coverage, complete with distribution mean (solid horizontal lines) and nominal level (dashed horizontal lines). As was the case for the functional components presented in Figure 7, nominal coverage of the scores is uniquely achieved by FAST. GFSR achieves lower mean coverages, particularly for the FPCs $\phi_1(t)$ at each level.

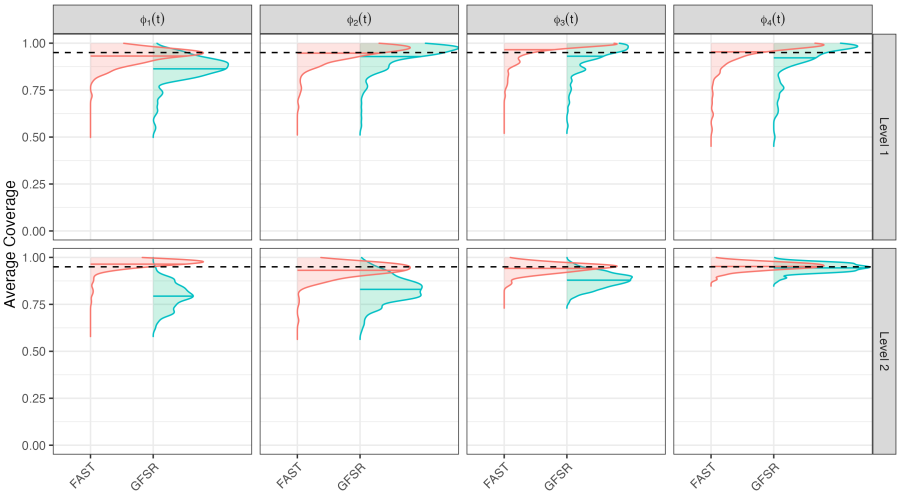

Figure 8: Kernel smoother of coverage probabilities of 95% credible intervals of the true scores, $\xi_{ik}^b, \zeta_{ijk}^b$, for FAST and GFSR from the multilevel simulations. Row indicates hierarchical level, while columns correspond to FPCs. The horizontal dotted lines indicate the nominal 95% level.

5 Simulation sensitivity analyses

For each sensitivity analysis, we vary one of the core hyper-parameters from the set K (number of FPCs), Q (dimension of the spline basis), and α (proportion of the penalty which is absolute rather than second order). The default values are $K = 3$ (the true number of FPCs), $Q = 20$, and $\alpha = 0.1$. All sensitivity analyses are conducted within simulation scenarios S1 and S2 described

in main text Section 4. We finally evaluate the effect of each of these sensitivity analyses on the computational efficiency of FAST in Section 5.4.

5.1 Chosen number of FPCs K

We first vary the fixed number of FPCs K which FAST estimates. The true value is $K = 3$ for scenarios S1 and S2, so we vary between $K = 2$ and $K = 6$ to understand the effects of insufficient and excessive K. We evaluate FAST under each K value using ISE and coverage of the FPCs as described in main text Section 4.

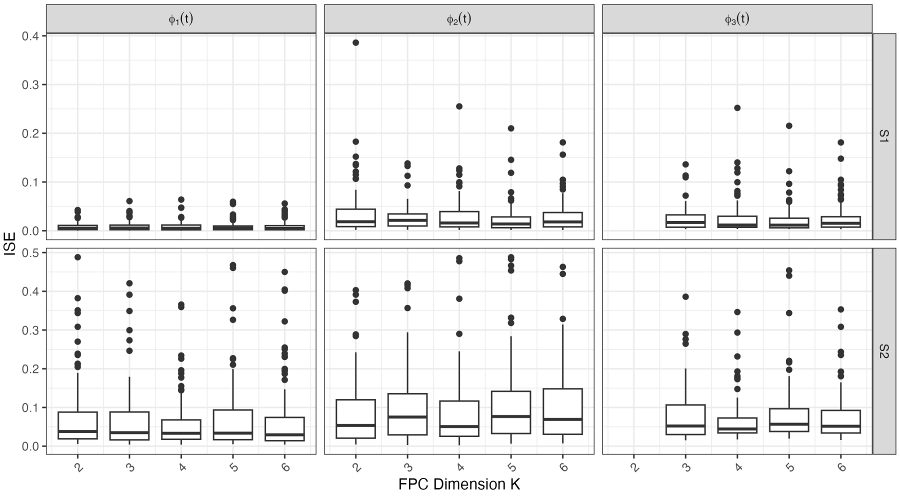

Figure 9: Boxplots of FPC ISE from applying FAST by number of chosen FPCs K. Columns indicate FPC, and rows correspond to simulation scenario.

Figure 9 indicates that the value of K does not appear to have a substantial impact on the accuracy of FPC estimates, outside of not estimating FPCs when they are present. When $K = 2$, the first two FPCs are still well estimated, and all 3 true FPCs are recovered with qualitatively similar accuracy whenever $K \geq 3$.

Figure 10 shows that $K = 2$ produces non-optimal inference for $\phi_1(t), \phi_2(t)$, with inferences for the first 3 FPCs unaffected when $K \geq 3$.

5.2 Chosen spline dimension Q

We next vary the spline basis dimension Q used by FAST to estimate the functional components of FPCA. We vary between $Q = 5$ and $Q = 40$ to understand the effects of both having a rather restrictive basis as well as having one which is very rich. We evaluate FAST under each Q value

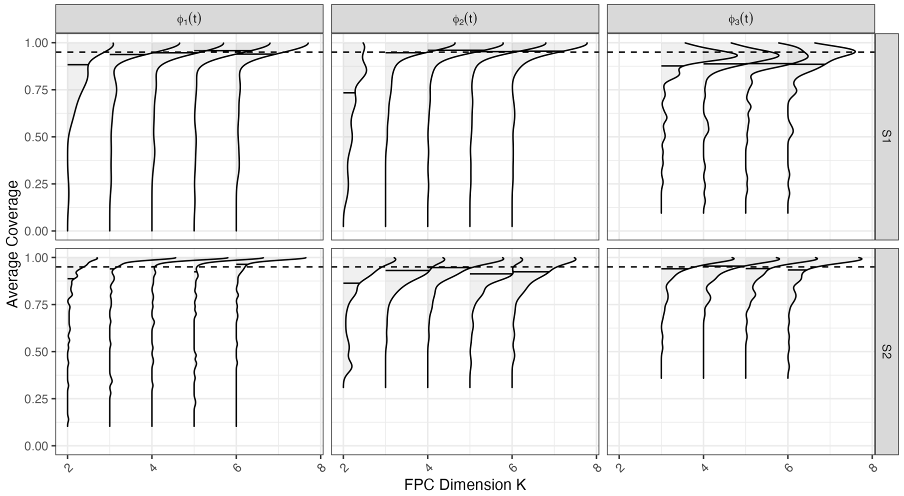

Figure 10: Kernel smooths of true FPC 95% credible interval coverage probabilities for FAST by number of chosen FPCs K. Columns indicate FPC, and rows correspond to simulation scenario.

using ISE and coverage of the FPCs as described in main text Section 4.

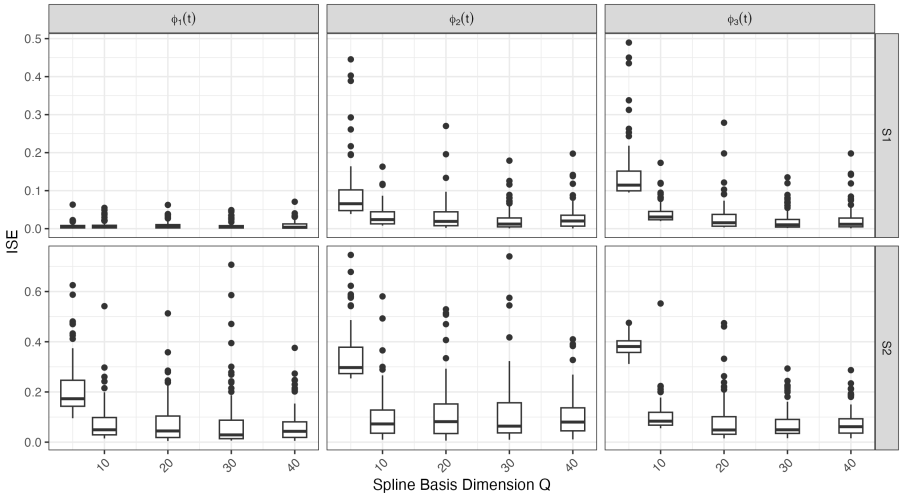

Figure 11: Boxplots of FPC ISE from applying FAST by spline basis dimension Q. Columns indicate FPC, and rows correspond to simulation scenario.

Figure 11 indicates setting $Q = 5$ produces biased estimates of the FPCs, likely due to lack of flexibility in the basis. However, the FPC ISE uniformly reduces and stabilizes by $Q = 20$ across both simulation scenarios.

Figure 12 similarly shows that $Q < 20$ produces non-optimal inference, with coverage stabilizing at near nominal levels for $Q \geq 20$.

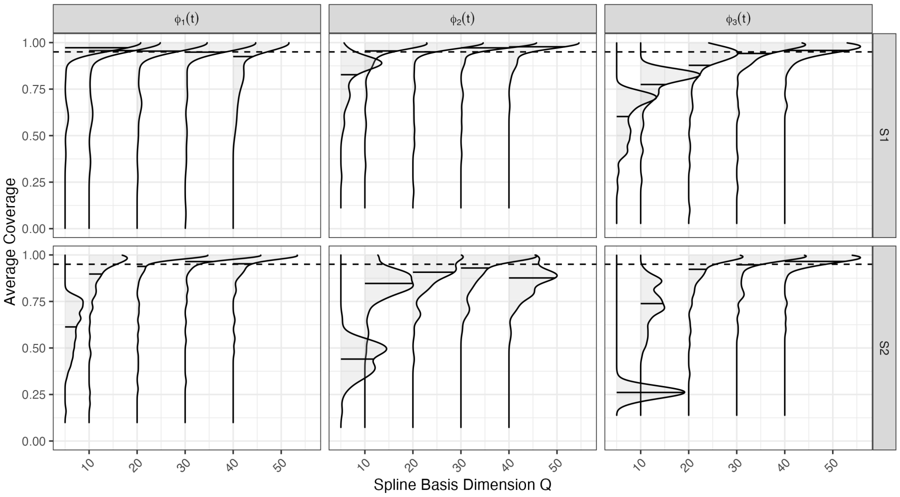

Figure 12: Kernel smooths of true FPC 95% credible interval coverage probabilities for FAST by spline basis dimension Q. Columns indicate FPC, and rows correspond to simulation scenario.

5.3 Chosen penalty parameter α

We finally vary penalty hyperparameter α, which dictates what proportion of the smoothing penalty used by FAST will be allocated to absolute value rather than second-order variation. We vary between $\alpha = 0.01$ and $\alpha = 0.3$, keeping $\alpha < 0.5$ to ensure the majority of the penalty is focused on the "wiggliness" which should be the core of any smoothing penalty. We evaluate FAST under each α value using ISE and coverage of the FPCs as described in main text Section 4.

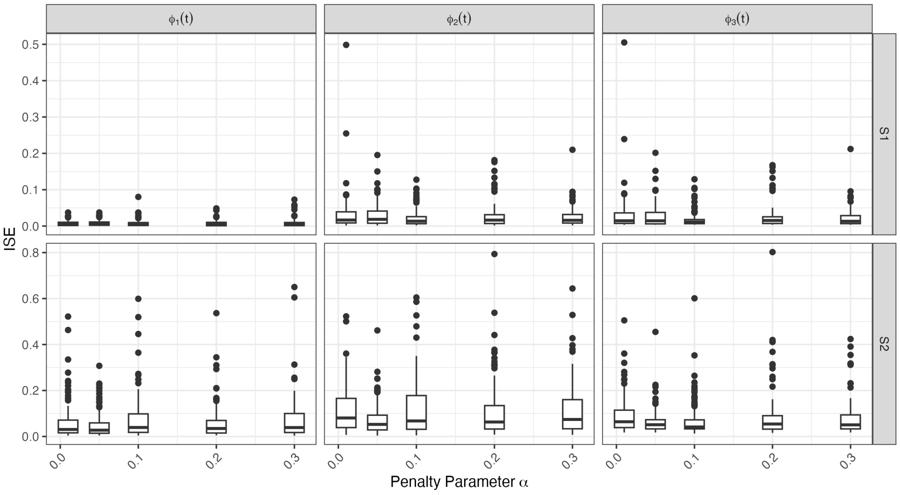

Figure 13: Boxplots of FPC ISE from applying FAST by penalty parameter α. Columns indicate FPC, and rows correspond to simulation scenario.

Figure 13 indicates that estimation accuracy, evaluated using ISE between the estimated and true FPCs, is consistent across the range of α values tested.

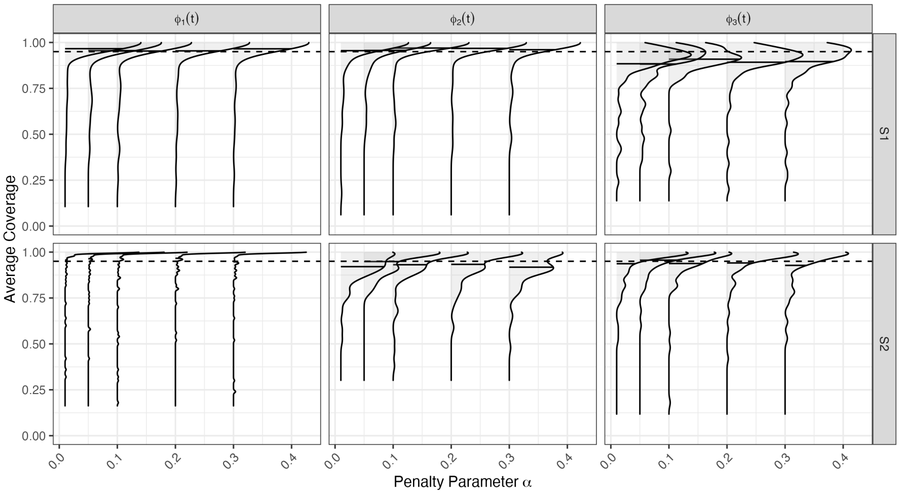

Figure 14: Kernel smooths of true FPC 95% credible interval coverage probabilities for FAST by penalty parameter α. Columns indicate FPC, and rows correspond to simulation scenario.

Figure 14 indicates that inference, evaluated using point-wise coverage of the true FPCs by equal-tailed 95% credible intervals, is also consistent across the range of α values tested.

5.4 Timing by Q, K

Given that it appears that estimation accuracy and inference are consistent when K and Q are sufficiently large, we evaluate the effect of increasing these quantities on the computation time of FAST. For this experiment, we focus on S1, the more computationally complex simulation scenario based upon CGM data. For each combination of $K \in \{3, 4, 5\}$ and $Q \in \{20, 30, 40\}$, we timed FAST on the same personal laptop described in main text Section 4 (2023 MacBook Pro with Apple M2 Max@3.49 GHz and 32GB of memory).

K/Q	$Q = 20$	$Q = 30$	$Q = 40$
$K = 3$	2.4	2.4	3.6
$K = 4$	2.2	2.1	2.7
$K = 5$	1.9	2.0	2.5

Table 1: Table of FAST BayesFPCA computation times (in minutes) for each combination of spline dimension Q and number of FPCs K. We fix the simulation to S1, number of time series to $N = 50$, and number of observations along the domain to $M = 50$.

From Table 1, we find that FAST does not require appreciable additional computation time when Q, K are increased.

6 Additional CGM Analyses/Details

6.1 Variability explained - Single Level

We plot the variability explained versus the number of FPCs used, K, for each of the 4 sub-diets in Figure 15. These values are calculated using frequentist FACE estimates of FPCA for each K value.

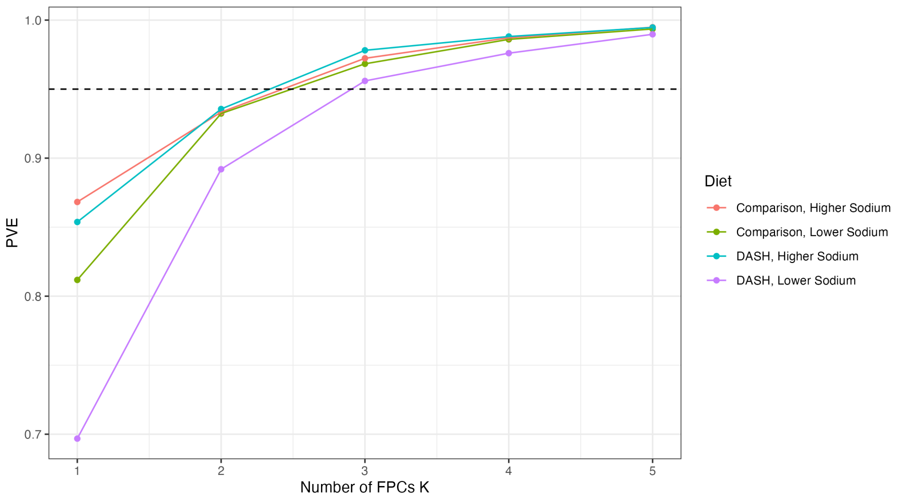

Figure 15: Variability in the single-level mean CGM curve data explained by FPCA approximation over a range of K values, stratified by diet.

Figure 15 indicates that, for all diets, $K = 3$ principal components explains more than 95% of the variability.

6.2 Bayesian FPCA of randomly-chosen CGM within diets

As an alternative single-level analysis, we randomly sample a function for each participant within each diet rather than aggregating. These functions each represent a single instantiation of the meal process for that participant within the particular diet, whereas the aggregate functions analyzed in main text Section 5.2 have varying levels of noise based upon the number of curves included in the average. However, when performing the by-diet FPCA analyses on these randomly chosen curves instead of the aggregated CGM, we observe qualitatively identical results. We first present the eigenfunctions with associated uncertainty in Figure 16. The primary difference we observe is in the additional sampling variability of $\widehat{\phi}_1(t)$ for the DASH, Lower Sodium diet, along with some of the posterior samples having greater curvature. This is indicative of

differences in mean mealtime glucose being much lower for this population, reduced to be on similar scale to differences peakedness.

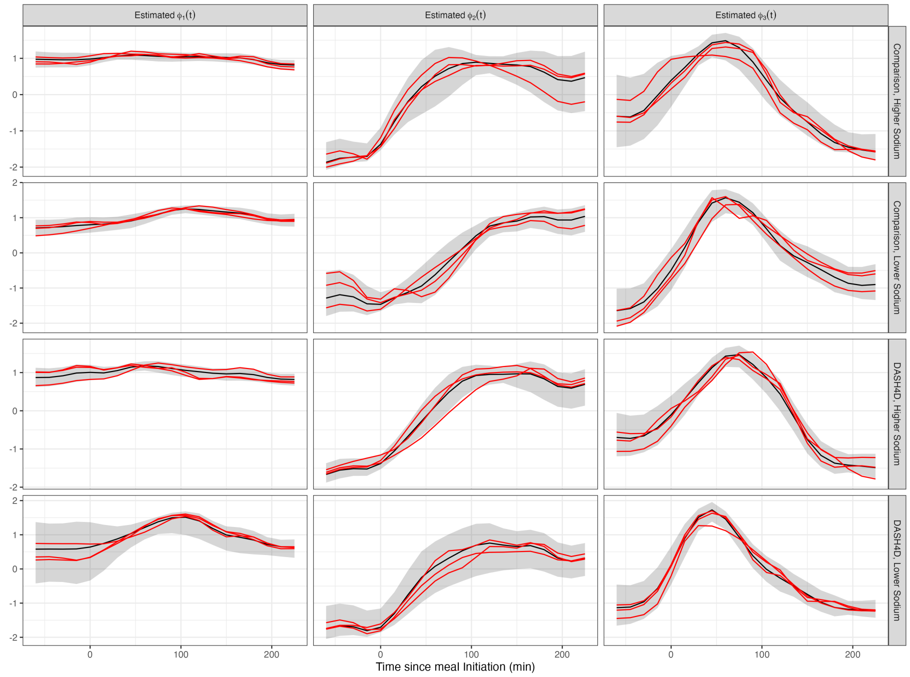

Figure 16: Bayesian FPCA results for the first three PCs (each column corresponds to one FPC) for each of the four diets (each row corresponds to one diet). X-axis: time from the start of the meal. Black curves: posterior mean; red curves: three samples from the posterior of the PCs; shaded areas: pointwise 95% credible intervals.

We present also the corresponding eigenvalues in Figure 17, where again one observes the unique properties of the DASH, Lower Sodium diet. In this analysis as well, there is lower overall variability for this diet (assessed by sum of eigenvalues). This difference is again driven by the first eigenvalue/eigenfunction pair in this analysis.

As for the aggregate data, we fit each model using 2000 iterations with the first 1000 discarded as burn-in. Employing the routine from main text Section 2.5 to assess convergence of the scores and FPCs, we found final Gelman-Rubin statistics were < 1.05.

References

Bagyan, A. and Richards, D. (2024). Complete Asymptotic Expansions for the Normalizing Constants of High-Dimensional Matrix Bingham and Matrix Langevin Distributions. *Symmetry, Integrability and Geometry: Methods and Applications*. arXiv:2402.08663 [math].

Bartle, R. (1995). Product Measures. In *The Elements of Integration and Lebesgue Measure*, pages 113–124. John Wiley & Sons, Ltd. Section: 10 _eprint: https://onlinelibrary.wiley.com/doi/pdf/10.1002/9781118164471.ch10.

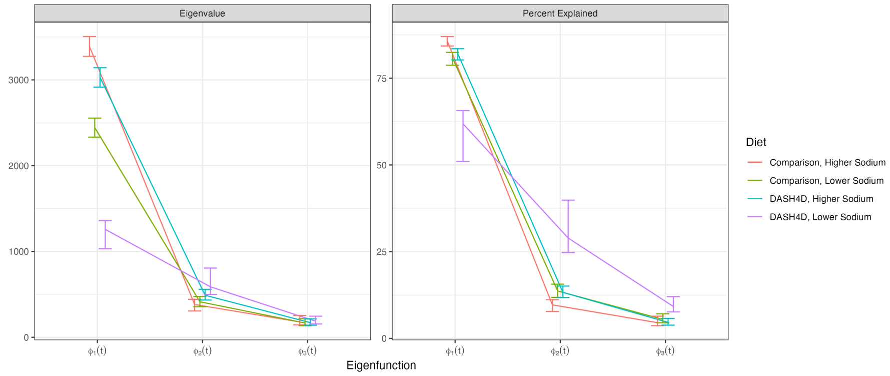

Figure 17: Eigenvalue and percent-variability estimates from the Bayesian FPCA models fit to each of the four diets (each line and color corresponds to one diet). X-axis: eigenfunction corresponding to eigenvalue/percent variance explained. All estimates are presented with their corresponding 95% credible intervals.

Craven, P. and Wahba, G. (1979). Smoothing noisy data with spline functions. *Numerische Mathematik*, 1:377–403.

Cui, E., Li, R., Crainiceanu, C. M., and Xiao, L. (2023). Fast Multilevel Functional Principal Component Analysis. *Journal of computational and graphical statistics : a joint publication of American Statistical Association, Institute of Mathematical Statistics, Interface Foundation of North America*, 32(2):366–377.

Faraut, 1940, J. and Korányi, A. (1994). *Analysis on symmetric cones*. Oxford : Clarendon Press ; New York : Oxford University Press, England, United Kingdom.

Goldsmith, J., Zipunnikov, V., and Schrack, J. (2015). Generalized Multilevel Function-on-Scalar Regression and Principal Component Analysis. *Biometrics*, 71(2):344–353.

Kimeldorf, G. and Wahba, G. (1970). A correspondence between bayesian estimation on stochastic processes and smoothing by splines. *The Annals of Mathematical Statistics*, 41(2):495–502.

Liu, X., Nassar, H., and PodgÓrski, K. (2020). Splinets – efficient orthonormalization of the B-splines. arXiv:1910.07341.

O'Sullivan, F. (1986). A statistical perspective on ill-posed inverse problems (with discussion). *Statistical Science*, 1(4):505–527.

Team, S. D. (2025). Stan modeling language users guide and reference manual 2.36.

Wahba, G. (1983). Bayesian "Confidence Intervals" for the Cross-Validated Smoothing Spline. *Journal of the Royal Statistical Society: Series B*, 45(1):133–150.
